# Supplementary material for: ACLY inhibition promotes tumour immunity and suppresses liver cancer
Source: Nature. 2025 Jul 30;645(8080):507–17. doi: 10.1038/s41586-025-09297-0 (PMC12422966; doi:10.1038/s41586-025-09297-0)
Supplement: Supplementary file 1 — This file contains Chemical supplementary Information of EVT0185 and EVT0185 Co.A, Supplementary Tables 1–5 and Supplementary Figure 1 [file 41586_2025_9297_MOESM1_ESM.pdf]

nature portfolio

https://doi.org/10.1038/s41586-025-09297-0

Supplementary information

ACLY inhibition promotes tumour immunity and suppresses liver cancer

In the format provided by the authors and unedited

SUPPLEMENTAL MATERIAL

6-[4-(5-Carboxy-5-methylhexyl)-phenyl]-2,2-dimethylhexanoic acid (EVT0185)

and

6-[4-(5-Carboxy-5-methylhexyl)-phenyl]-2,2-dimethylhexanoic Acid Coenzyme A Ester (EVT0185-CoA)

Syntheses and Characterization

General information

Reagents were purchased from Sigma-Aldrich, SynQuest Labs or Oakwood Chemical. Testing agents EVT0185 and EVT0185-CoA were prepared at Symeres (Kerkenbos 1013, 6546 BB Nijmegen, The Netherlands, www.symeres.com).

Synthesis of 6-[4-(5-Carboxy-5-methylhexyl)-phenyl]-2,2-dimethylhexanoic Acid (EVT0185)

The compound was synthesized according to **Scheme 1**.

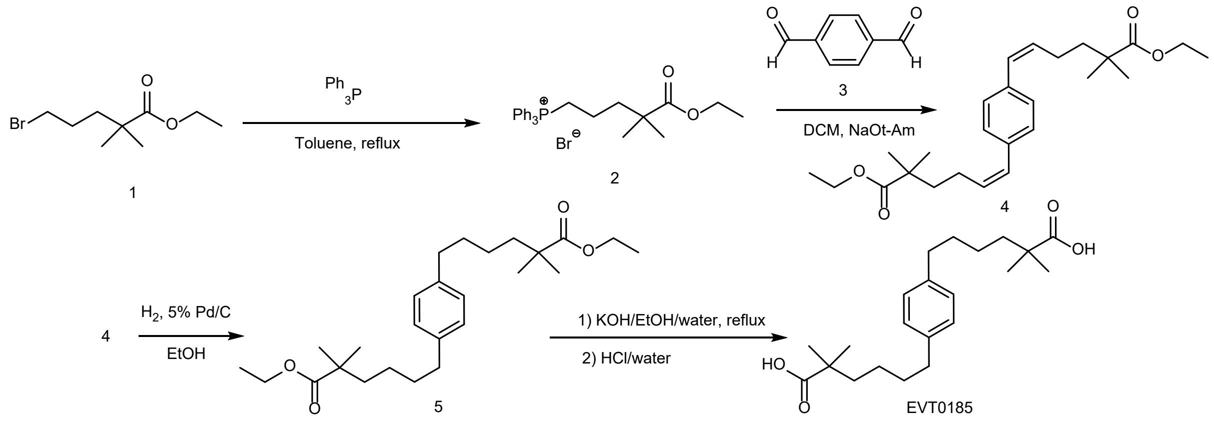

Scheme 1

Experimental Part

(5-Ethoxy-4,4-dimethyl-5-oxopentyl)(triphenyl)phosphanium bromide (2)

Triphenylphosphine (1553.0 g, 5.921 mol) was added to a solution of 5-bromo-2,2-dimethylpentanoic acid ethyl ester **1** (1404.0 g, 5.921 mol) in toluene (11 L). The solution was heated to reflux for 48 h. After cooling to room temperature overnight without stirring, the toluene was decanted and saved. The residue was used in the next step without further purification. The procedure generated a first crop of intermediate **2**. The decanted toluene was concentrated, the remaining residue (~668g) was mixed with toluene (650 mL) and heated to reflux under argon for 30 hours. After 30 hours, the mixture was cooled to room temperature and let to stand overnight. After 20 hours, the toluene was decanted, and the remaining residue was dissolved in dichloromethane (550 mL). The resulting solution was evaporated and the remaining solid was dried under vacuum in a rotary evaporator (rotovap) to a constant weight.

The procedure generated a second crop of intermediate **2**. After the crops were combined, the experiment generated intermediate **2** (2773.0 g, 94% yield) as an off-white solid.

^{1}H NMR (300 MHz, $CDCl_3$): δ (ppm) 7.89-7.65 (m, 15H), 3.96 (q, 2H, *J* = 7.2 Hz), 3.91-3.81 (m, 2H), 1.94-1.87 (m, 2H), 1.65-1.54 (m, 2H), 1.10 (t, *J* = 7.2 Hz, 9H).

^{31}P (121 MHz, $CDCl_3$): δ (ppm) 24.3.

Ethyl (5E/Z)-6-{4-[(1E/Z)-5,5-dimethyl-6-ethoxy-6-oxohex-1-en-1-yl]phenyl}2,2-dimethyl-hex-5-enoate (4)

(5-Ethoxy-4,4-dimethyl-5-oxopentyl)(triphenyl)phosphanium bromide **2** (2050 g, 4.105 mol) and terephthalaldehyde **3** (220.2 g, 1.642 mol) were dissolved in DCM (9.6 L) at room temperature under an argon atmosphere. Sodium *tert*-amylate (474.5 g, 4.269 mol) was added in portions (10x). The flask was cooled in a water bath at room temperature to maintain the reaction temperature below 35 °C. After addition, the reaction mixture was vigorously stirred for 3 hours at room temperature. Deionized water (3.5 L) was added, and the mixture was neutralized with addition of 1N HCl (250 mL). The layers were separated, and the aqueous fraction was extracted with dichloromethane (1.8 L). The dichloromethane phases were combined, dried over magnesium sulfate, filtered, and concentrated under vacuum. To the residue (2362 g) was added MTBE (2 L), and the resulting suspension was rotated at 48 °C for 0.5 h, then at 2-9 °C for 1 h. The resulting solid was filtered and washed with MTBE (2 x 1L). The filtrate was concentrated. The remaining yellow oil (1076 g) was purified by column chromatography on silica gel (9.6 kg) eluting with 2% to 5% ethyl acetate in heptane. The procedure generated an E/Z mixture of isomers of intermediate **4** (598.8 g, 88% yield), as a light-yellow oil. HPLC purity: 99.6% (UV detection, 270 nm).

^{1}H NMR (300 MHz, $CDCl_3$): δ (ppm) 7.29-7.16 (m, 4H), 6.40-6.32 (m, 2H), 6.22-6.12 (m, 1H), 5.63-5.52 (m, 1H), 4.16-4.05 (m, 4H), 2.33-2.10 (m, 4H), 1.75-1.62 (m, 4H), 1.28-1.12 (m, 18H).

6-[4-(5-Carboxy-5-methylhexyl)-phenyl]-2,2-dimethylhexanoic acid diethyl ester (5)

Intermediate **4** (582.8 g, 1.406 mol) was dissolved in ethanol (3 L) and added to 5% palladium on carbon (100 g) under an argon atmosphere at room temperature. The argon atmosphere was replaced with hydrogen gas (50-60 psi) and the mixture was hydrogenated on a Parr hydrogenator for 5 hours at room temperature. After 5 hours, the hydrogen was replaced with nitrogen and the mixture was diluted with ethyl acetate (2 L) and then filtered through a

membrane. The catalyst was washed with ethyl acetate (2 x 0.5 L). The filtrate and washings were concentrated under vacuum in a rotovap and the crude material was used for the final step without purification. The procedure generated intermediate **5** (585.7 g, 99% yield) as a colorless oil. HPLC purity: 98.3% (UV detection, 270 nm).

^{1}H NMR (300 MHz, $CDCl_3$): δ (ppm) 7.06 (s, 4H), 4.09 (q, 4H, *J* = 7.2 Hz), 2.55 (t, 4H, *J* = 7.8 Hz), 1.63-1.50 (m, 8H), 1.32-1.08 (m, 4H), 1.22 (t, 6H, *J* = 7.2 Hz), 1.15 (s, 12H).

6-[4-(5-Carboxy-5-methylhexyl)-phenyl]-2,2-dimethylhexanoic acid (EVT0185)

Intermediate **5** (1100.7 g, 2.629 mol) was dissolved in ethanol (8 L). Di water (7 L) containing potassium hydroxide (1025.7 g, 86%, 15.776 mol) was added and the mixture was heated to reflux under an argon atmosphere. After 6 hours, the heat was turned off and the mixture cooled to room temperature and stirred overnight. After 18 hours, the solution was concentrated on a rotovap to remove the ethanol. The remaining mixture was diluted with DI water (7 L), and extracted with MTBE (2 x 3.5L). The aqueous portion was acidified (to pH < 1) with concentrated hydrochloric acid (2730 g) in DI water (2.73 L) (added at 40-50 °C). The resulting suspension was stirred for 1 hour at room temperature. Ethyl acetate (10 L) was added, and the mixture was stirred for another hour. The phases were separated, and the aqueous phase was extracted with ethyl acetate (2 × 5 L). The combined ethyl acetate extracts were washed with water (5 L) followed by brine (3 L), dried over magnesium sulfate, filtered, and concentrated under vacuum in a rotavap until solid precipitated. Heptane (2 L) was added and rotated in a rotavap at 45 °C until a clear solution formed. Then the clear solution was evaporated to dryness and dried at 45 °C on a rotovap until constant weight. The procedure generated 6-[4-(5-carboxy-5-methylhexyl)-phenyl]-2,2-dimethylhexanoic acid **EVT0185** (934.0 g, 98% yield, 99.0% purity by HPLC, rt = 11.05 min, by UV detection, 220 nm) as a white solid, m.p.: 125-126 °C. HRMS (m/z): $C_{22}H_{34}O_4$, [M-H]- Calculated: 361.2384; found: 361.2383.

^{1}H NMR (300 MHz, $CDCl_3$): δ (ppm) 7.08 – 6.98 (m, 4H), 2.60 – 2.49 (m, 4H), 1.63 – 1.46 (m, 8H), 1.34 – 1.21 (m, 4H), 1.13 (s, 12H).

^{13}C NMR (75 MHz, $CDCl_3$): δ (ppm) 179.25, 138.01, 126.32, 40.07, 38.82, 33.39, 30.36, 22.76.

Synthesis of 6-[4-(5-Carboxy-5-methylhexyl)-phenyl]-2,2-dimethylhexanoic Acid Coenzyme A Ester (EVT0185-CoA)

The compound was synthesized according to **Scheme 2**.

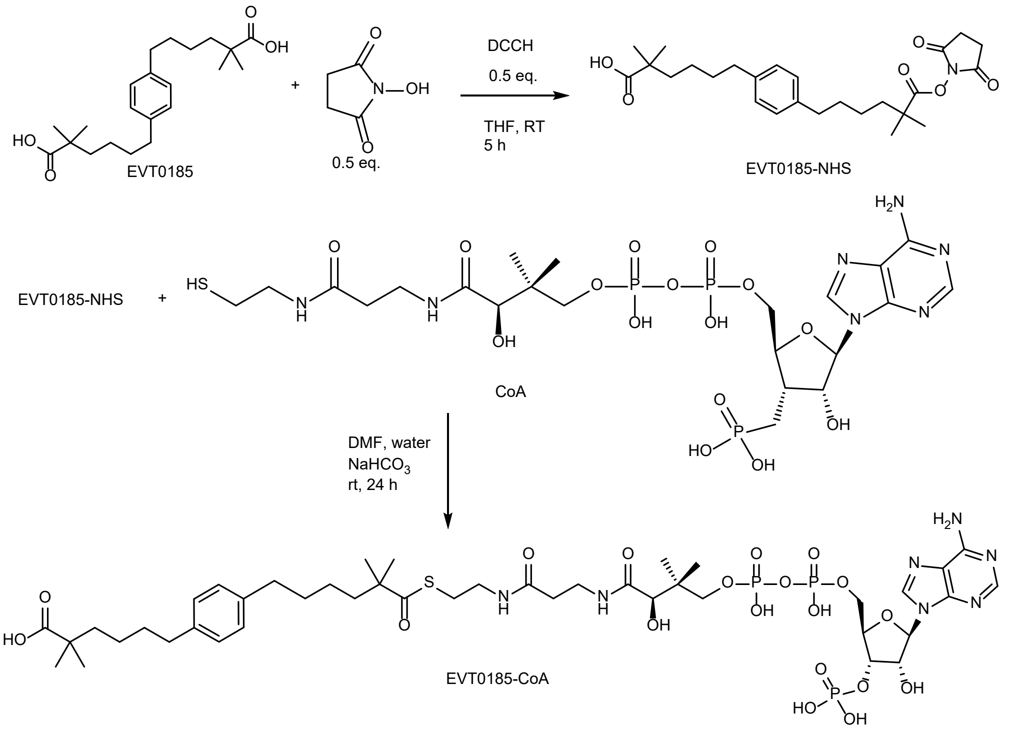

Scheme 2: Synthesis of and 6-[4-(5-carboxy-5-methylhexyl)-phenyl]-2,2-dimethylhexanoic acid Coenzyme A ester (**EVT0185-CoA**)

Experimental Part

6-(4-(6-((2,5-Dioxopyrrolidin-1-yl)oxy)-5,5-dimethyl-6-oxohexyl)phenyl)-2,2-dimethylhexanoic acid (EVT0185-NHS)

A mixture of EVT0185 (2.0 g, 5.51 mmol), N-hydroxysuccinimide (300 mg, 2.6 mmol), and dicyclohexylcarbodiimide (DCC, 580 mg, 2.8 mmol) were stirred for 5 hours in THF (35 mL) at room temperature under an argon atmosphere. The reaction mixture was filtered to remove dicyclohexylurea (DCU) and was concentrated under vacuum on a rotary evaporator. The half-ester EVT0185-NHS was purified by column chromatography on silica gel (60 g) eluting with 20%-50% ethyl acetate/heptane. The procedure generated EVT0185-NHS (0.92 g, 77% yield) as a white solid.

^1H NMR (300 MHz, $CDCl_3$): δ (ppm) 7.08 (s, 4H), 2.82 (m, 4H), 2.58 (q, 4H, J= 7.2 Hz), 1.74-1.56 (m, 8H), 1.51-1.38 (m, 4H), 1.34 (s, 6H), 1.19 (s, 6H).

6-[4-(5-carboxy-5-methylhexyl)-phenyl]-2,2-dimethylhexanoic acid Coenzyme A ester (EVT0185-CoA: 6-(4-(6-((2-(3-((2R)-4-(((((((2R,3S,4R,5R)-5-(6-Amino-9H-purin-9-yl)-4-hydroxy-3-(phosphonooxy)tetrahydrofuran-2-yl)methoxy)(hydroxy)phosphoryl)oxy)-(hydroxy)phosphoryl)oxy)-2-hydroxy-3,3-dimethylbutanamido)propanamido)ethyl)thio)-5,5-dimethyl-6-oxohexyl)phenyl)-2,2-dimethylhexanoic acid)

Water used as a solvent was degassed with argon prior to usage. A solution of 6-(4-(6-((2,5-dioxopyrrolidin-1-yl)oxy)-5,5-dimethyl-6-oxohexyl)phenyl)-2,2-dimethylhexanoic acid (2.41 g, 5.24 mmol) in Acetone (50 ml) was cooled with ice water after which a solution of CoA trilithium salt (1.030 g, 1.311 mmol) in Water (5 ml) was added, followed by a solution of sodium bicarbonate (1.498 g, 17.83 mmol) in Water (20 ml). The ice bath was removed after about 5 minutes and the reaction mixture was stirred under argon atmosphere at room temperature for 5 days. Acetone was removed from the reaction mixture *in vacuo* using a rotary evaporator setup without using the water bath resulting in a thick suspension. The aqueous reaction mixture was washed 4 times with Et2O while diluting with some additional water. The aqueous layer was freeze dried overnight affording 2.23 g off white powder which was dissolved in 10-14 ml of water and purified in 3 batches by basic preparative reversed phase chromatography. Appropriate fractions were combined and concentrated by lyophilization affording 386 mg (purity >85%, 0.347 mmol, yield: 27%) of the desired product as a clear to slightly tan glass. Chromatographic purity LCMS: >85%, Mass:m/z [M-H]+ 1110.2. HRMS standard ESI conditions (m/z): $C_{43}H_{68}N_7O_{19}P_3S$, [M-H]- Calculated: 1110.3431; found: 1110.3440.

^1H NMR (300 MHz, DMSO-d6): δ (ppm) 8.45 (s, 1H), 8.13 (s, 1H), 7.93 (t, J = 6.1 Hz, 0.6H), 7.07 – 6.96 (m, 4H), 6.06 (d, J = 6.2 Hz, 1H), 4.53 – 4.45 (m, 1H), 4.20 – 4.08 (m, 2H), 3.94 (s, 1H), 3.76 (dd, J = 9.8, 5.0 Hz, 1H), 3.47 (dd, J = 9.8, 4.9 Hz, 1H), 3.34 (m, 2H), 3.13 (t, J = 6.4 Hz, 2H), 2.81 (t, J = 6.3 Hz, 2H), 2.48 – 2.37 (m, 4H), 2.30 (t, J = 6.7 Hz, 2H), 1.60 – 1.35 (m, 8H), 1.20 – 0.90 (m, 4H), 1.03 (s, 6H), 0.98 (s, 6H), 0.81 (s, 3H), 0.66 (s, 3H). One signal (2H) coincides with the HOD signal.

^{13}C NMR (100 MHz, [D_2O]/TMS): δ (ppm) 208.84, 183.65, 174.64, 173.21, 155.04, 152.20, 148.95, 139.69, 139.28, 128.07, 118.36, 86.61, 83.42, 74.07, 73.83, 71.88, 65.29, 49.60, 42.13, 41.73, 40.42, 40.28, 38.75, 38.36, 38.28, 35.43, 35.33, 34.92, 34.71, 31.81, 31.47, 27.48, 24.91, 24.78, 24.40, 23.83, 21.00, 17.93.

Raw Data

6-[4-(5-Carboxy-5-methylhexyl)-phenyl]-2,2-dimethylhexanoic Acid

EVT0185

ANALYTICAL REPORT – HRMS ANALYSIS

Symeres Project Code	:	SY21020222
Client	:	Espervita Therapeutics
Contact Person	:	Edward Bijsterveld
Author	:	Ilonka Meerts
Report Date	:	March 1, 2022
Reviewer	:	Juha Jyrkäs

1. Objective

In this study the exact mass of a compound is confirmed using the LC-MS Q Exactive Focus high resolution mass spectrometer (Thermo Scientific).

2. Procedure

2.1. Calibration

Before starting the analyses, the mass spectrometer was calibrated by infusion of a standard mixture using the syringe pump at 5 µl/min:

- Positive ion mode: Pierce calibration solution containing n-butylamine, caffeine, MRFA and Ultramark 1621 (Thermo Fisher Scientific product nr 88323).
- Negative ion mode: Pierce calibration solution containing sodium dodecyl sulfate, sodium taurocholate, and Ultramark 1621 (Thermo Fisher Scientific product nr 88324).

2.2. Analysis

The sample was dissolved in methanol with a concentration of 1 mg/ml and further diluted to a concentration of 10 µg/ml in acetonitrile. 1 µl sample was injected and analyzed using the HRMS method (for details, see Appendix). The data were acquired under full MS mode (resolution 70000 FWHM at 200 Da; < 3 ppm accuracy) over the mass range m/z of 150 – 2000 (negative mode). Standard ESI conditions compatible with the flow rate were applied: spray voltage 2.5 kV, auxiliary gas heater temperature 463°C, capillary temperature 281°C, sheath gas 58, auxiliary gas 16, sweep gas 3, S-lens RF level 50.

Data was evaluated using Xcalibur Qual Browser version 4.4.16 (Thermo Fisher).

info@symeres.com
+31(0)243723300

Kerkenbos 1013, 6546 BB Nijmegen
PO Box 6747, 6503 GE Nijmegen
The Netherlands

Trade Register Arnhem 09127273
VAT NL810510807B01

Mercachem B.V. and its affiliated companies are
operating under the registered trade name Symeres

3. EVT0185

HRMS (m/z): $C_{22}H_{34}O_4$, $[M-H]^-$ Calculated: 361.2384; found: 361.2383, Δppm -0.28.

Additional mass detected: 383.2202 corresponding to $[M+Na-2H]^-$ ion.

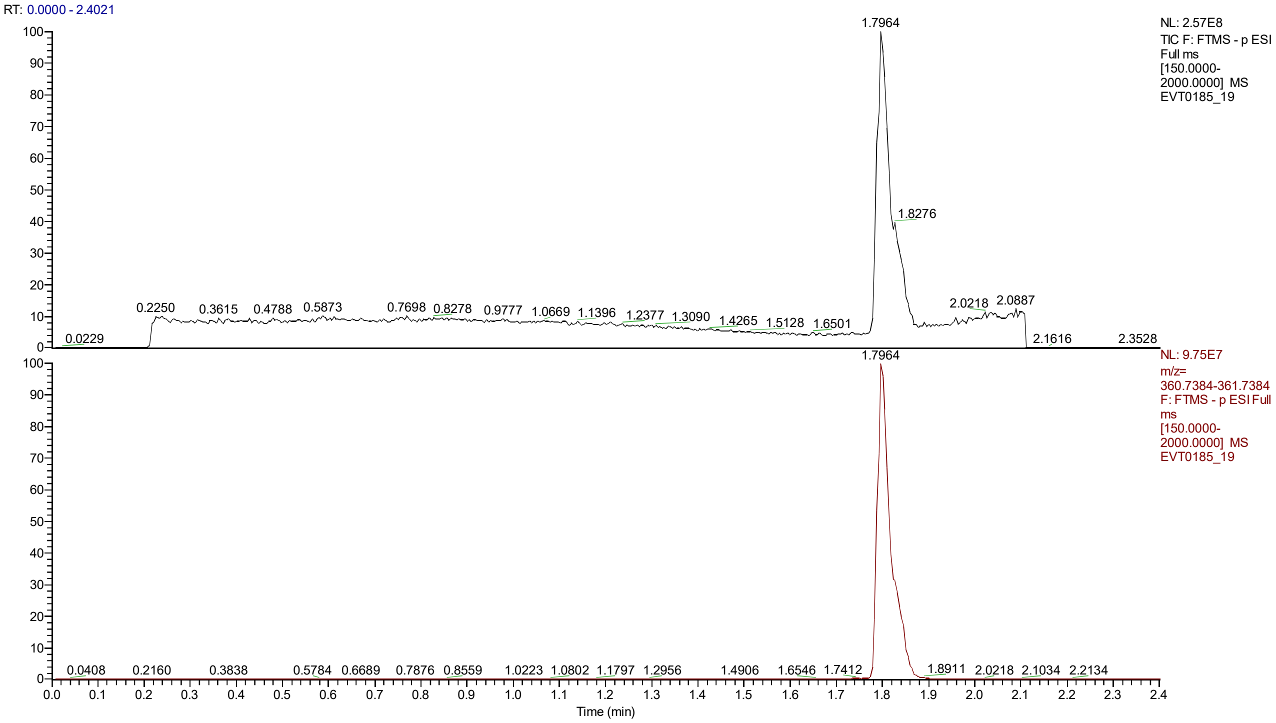

EVT0185: Top: TIC, Bottom: Extracted Ion Chromatogram of m/z 361.2384.

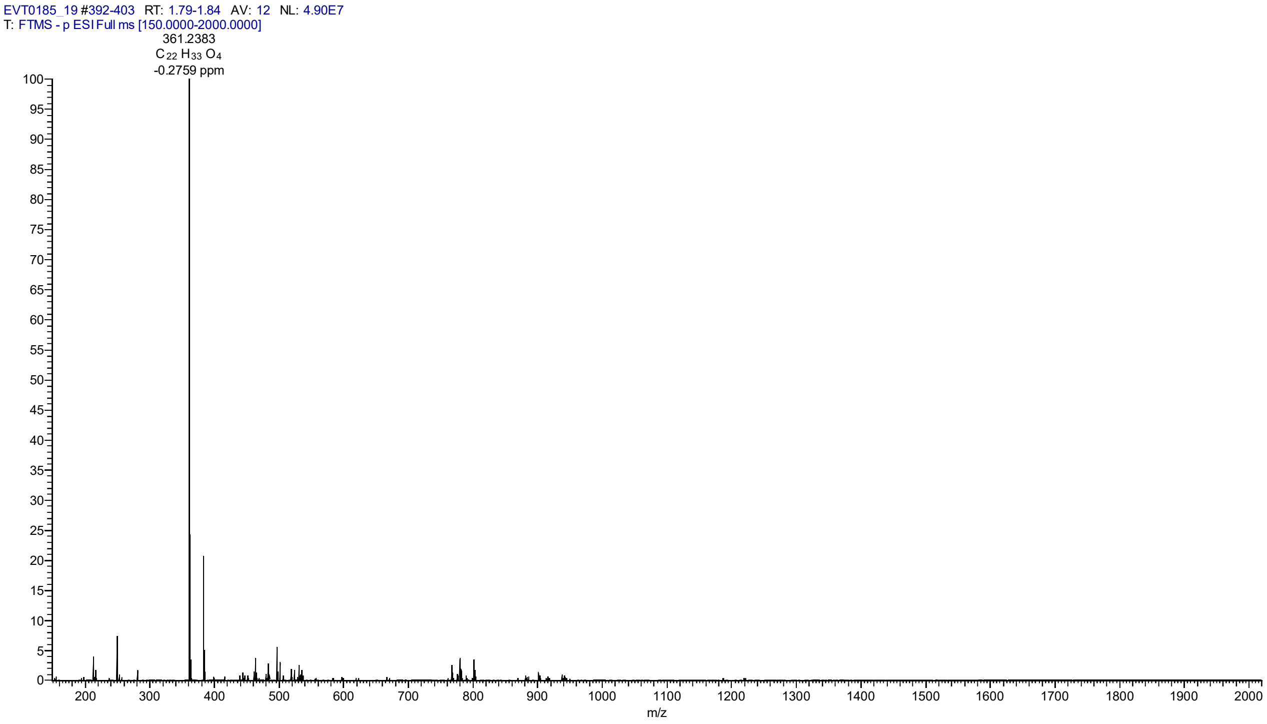

EVT0185: MS Spectrum @ 1.80 minutes.

www.symeres.com

Symeres

title	**STBE05-077-1**
Date acquired	24-Jan-2022, 15:07:04
FileName	Analysis\lcms20_20220124_220107
UPLC	Waters I-Class
Acq. Method	UPLC_AN_BASE
Column	XSelect CSH C18 XP (50x2.1mm 2.5µr
Flow	0.6 ml/min; Column temp: 25°C
Eluent A	10mM ammoniumbicarbonate in wa
Eluent B	acetonitrile
Gradient	t=0 min 5% B, t=2 min 98% B, t=2.7 mi
Posttime	0.3 min
Detection PDA	210-320nm
MS	QDa
Detection MS	ESI (scan)
Mass range	100-800 (pos)

Integrals spectrum PDA - Total Absorbance Chroma

rt (min)	height	area
0.87	29089155	501663808

PDA - Total Absorbance Chromatogram

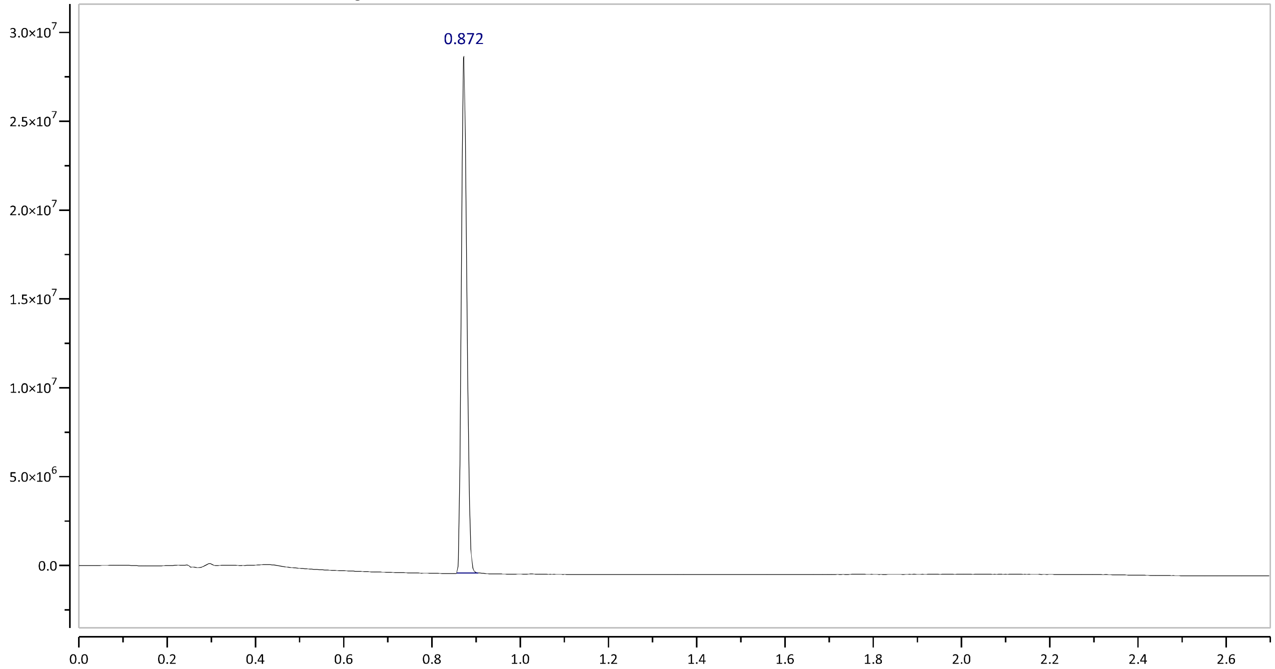

MS - spectrum 0.87

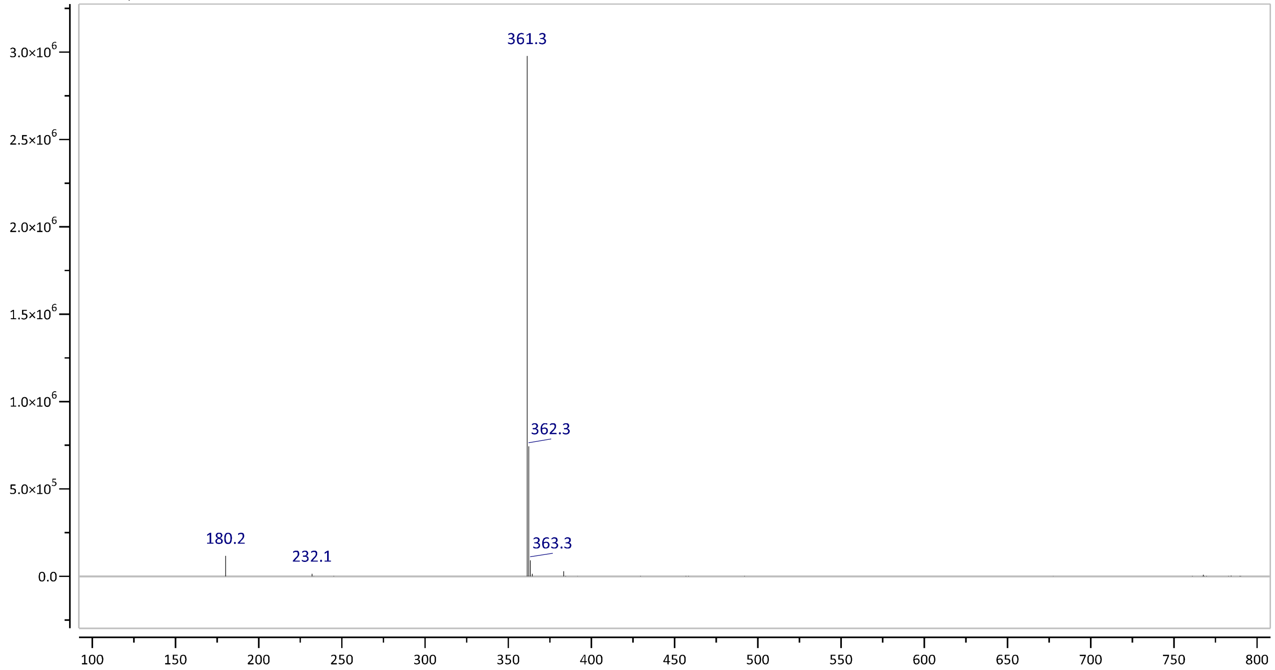

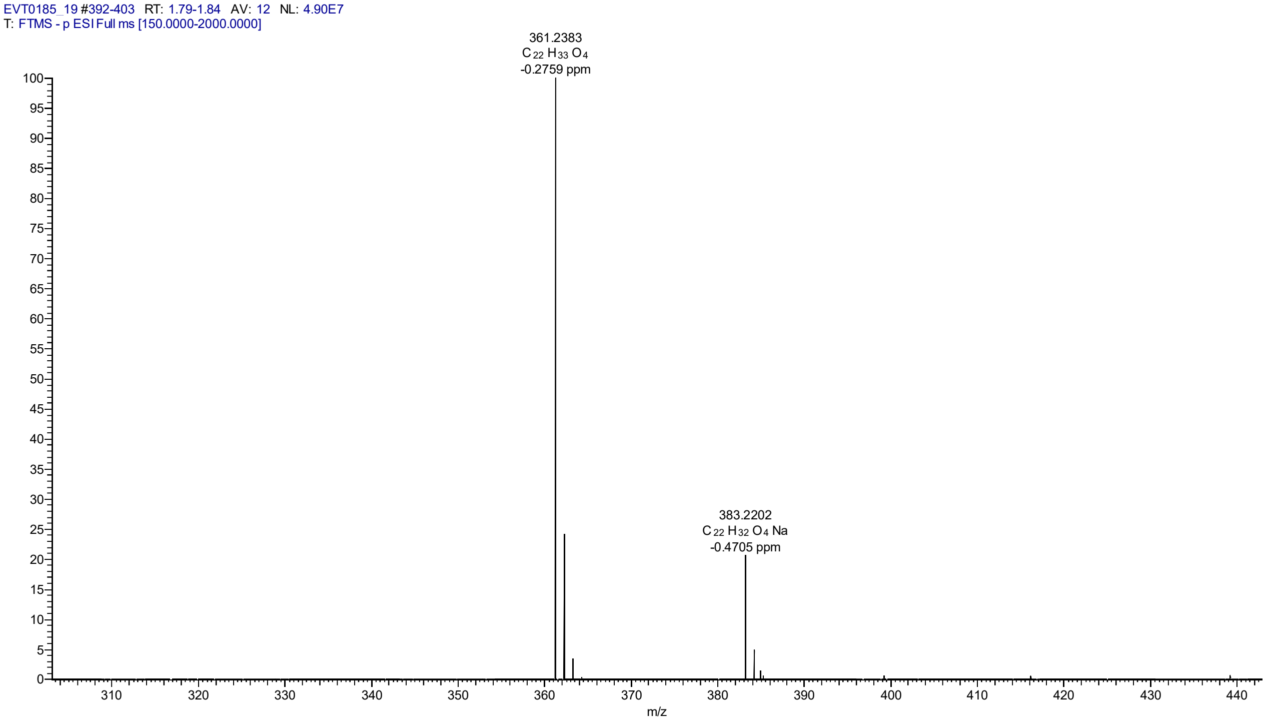

EVT0185: MS Spectrum @ 1.80 minutes, zoomed in.

www.symeres.com

Symeres

Title	**STBE05-077-1**
Data File Name	analyse_20220124_420_141840
Method	1D
Origin	Bruker BioSpin GmbH
Pulse Sequence	zg30
Relaxation Delay	1 s
Solvent	MeOD
Acquisition Date	24 Jan 2022 (14:18)
Number of Scans	16
Frequency	400 MHz
Nucleus	1H

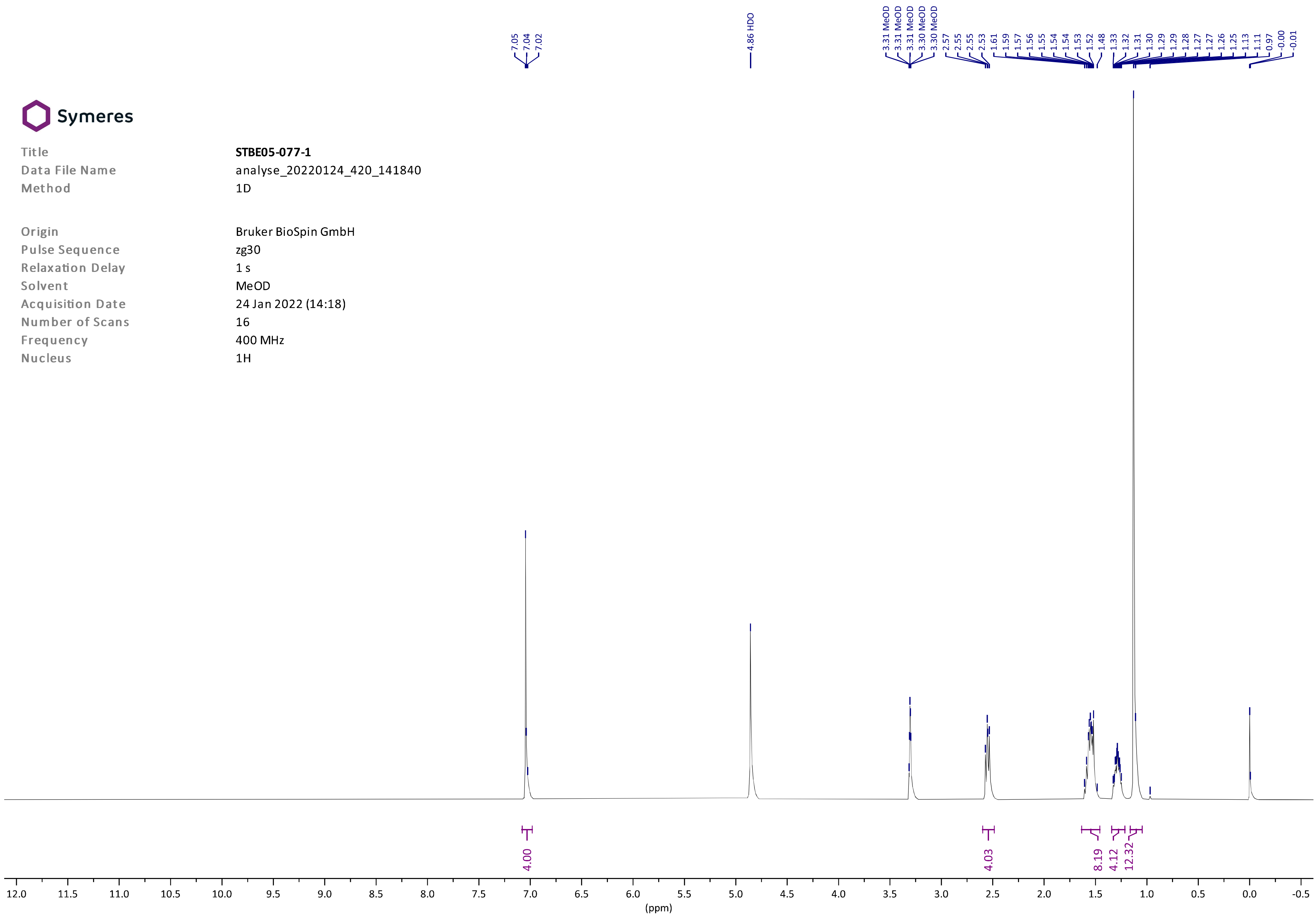

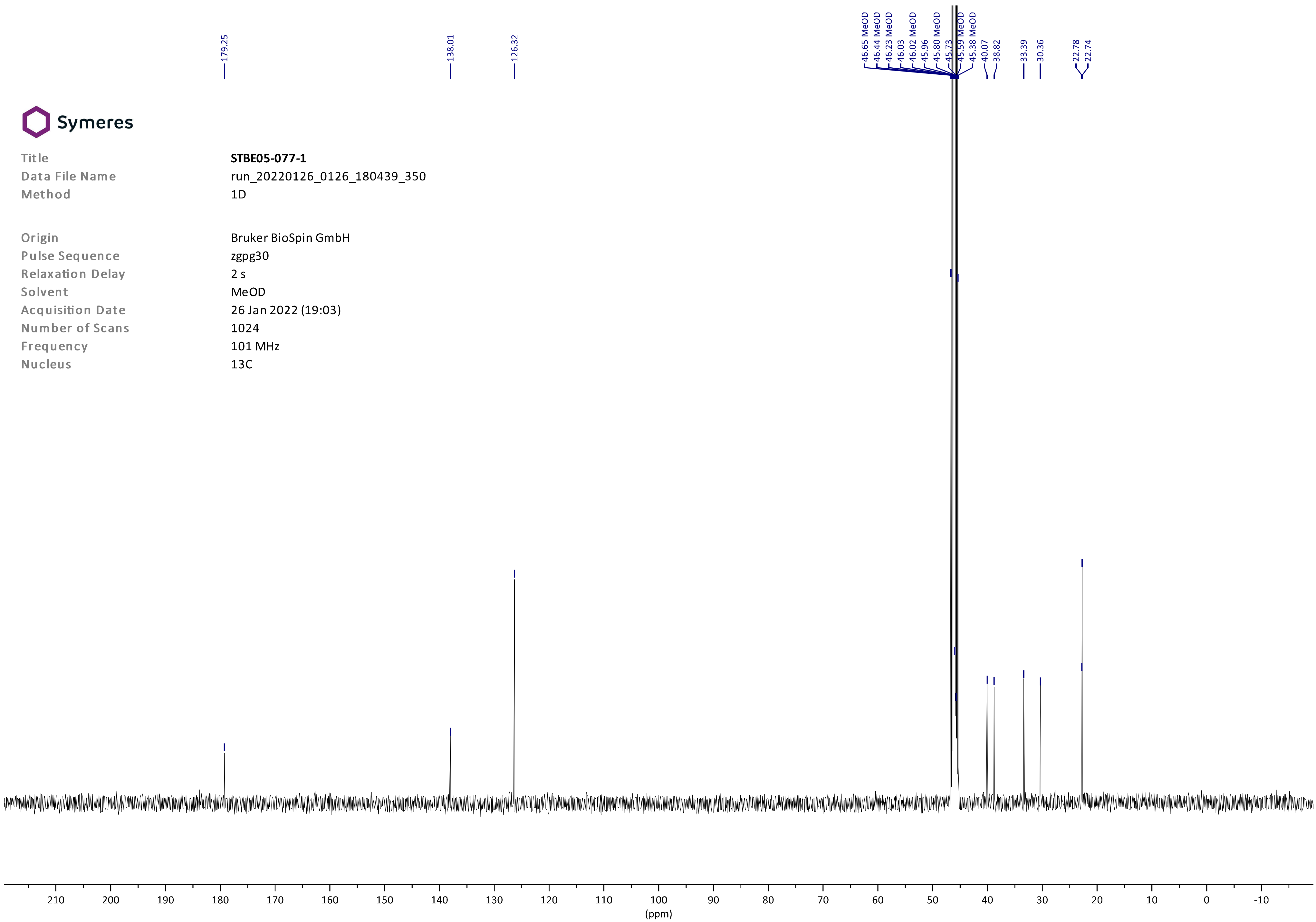

Symeres

Title	**STBE05-077-1**
Data File Name	run_20220126_0126_180439_350
Method	1D
Origin	Bruker BioSpin GmbH
Pulse Sequence	zgpg30
Relaxation Delay	2 s
Solvent	MeOD
Acquisition Date	26 Jan 2022 (19:03)
Number of Scans	1024
Frequency	101 MHz
Nucleus	13C

Raw Data

6-[4-(5-Carboxy-5-methylhexyl)-phenyl]-2,2-dimethylhexanoic Acid Coenzyme A Ester

EVT0185-CoA

ANALYTICAL REPORT – HRMS ANALYSIS

Symeres Project Code	:	SY21020222
Subject	:	HRMS22-011
Client	:	Espervita Therapeutics
Contact Person	:	Edward Bijsterveld
Author	:	Juha Jyrkäs
Report Date	:	April 4, 2022
Reviewer	:	Eef van den Elzen

1. Objective

In this study the exact mass of a compound is confirmed using the LC-MS Q Exactive Focus high resolution mass spectrometer (Thermo Scientific).

2. Procedure

2.1. Calibration

Before starting the analyses, the mass spectrometer was calibrated by infusion of a standard mixture using the syringe pump at 5 µl/min:

- Positive ion mode: Pierce calibration solution containing n-butylamine, caffeine, MRFA and Ultramark 1621 (Thermo Fisher Scientific product nr 88323).
- Negative ion mode: Pierce calibration solution containing sodium dodecyl sulfate, sodium taurocholate, and Ultramark 1621 (Thermo Fisher Scientific product nr 88324).

2.2. Analysis

A solution of the sample in acetonitrile with a concentration of 10 µg/ml was prepared. 1 µl sample was injected and analyzed using the HRMS method (for details, see Appendix). The data were acquired under full MS mode (resolution 70000 FWHM at 200 Da; < 3 ppm accuracy) over the mass range m/z of 150 – 2000 (negative mode). Standard ESI conditions compatible with the flow rate were applied: spray voltage 2.5 kV, auxiliary gas heater temperature 463°C, capillary temperature 281°C, sheath gas 58, auxiliary gas 16, sweep gas 3, S-lens RF level 50.

Data was evaluated using Xcalibur Qual Browser version 4.4.16 (Thermo Fisher).

info@symeres.com
+31(0)243723300

Kerkenbos 1013, 6546 BB Nijmegen
PO Box 6747, 6503 GE Nijmegen
The Netherlands

Trade Register Arnhem 09127273
VAT NL810510807B01

Mercachem B.V. and its affiliated companies are
operating under the registered trade name Symeres

3. Results

Sample name	Formula	Ion	Calculated m/z	Found m/z	Δppm
GELI73-012-1	C43H68N7O19P3S	[M-H]-<br>[M-2H]2-	1110.3431<br>554.6679	1110.3445<br>554.6683	1.29<br>0.71
GELI73-015-5	C43H68N7O19P3S	[M-H]-<br>[M-2H]2-	1110.3431<br>554.6679	1110.3445<br>554.6683	0.80<br>0.14

3.1. GELI73-012-1

HRMS (m/z): C43H68N7O19P3S, [M-H]-, Calculated: 1110.3431; found: 1110.3445, Δppm: 1.29, [M-2H]2-, Calculated: 554.6679, found 554.6683, Δppm: 0.71

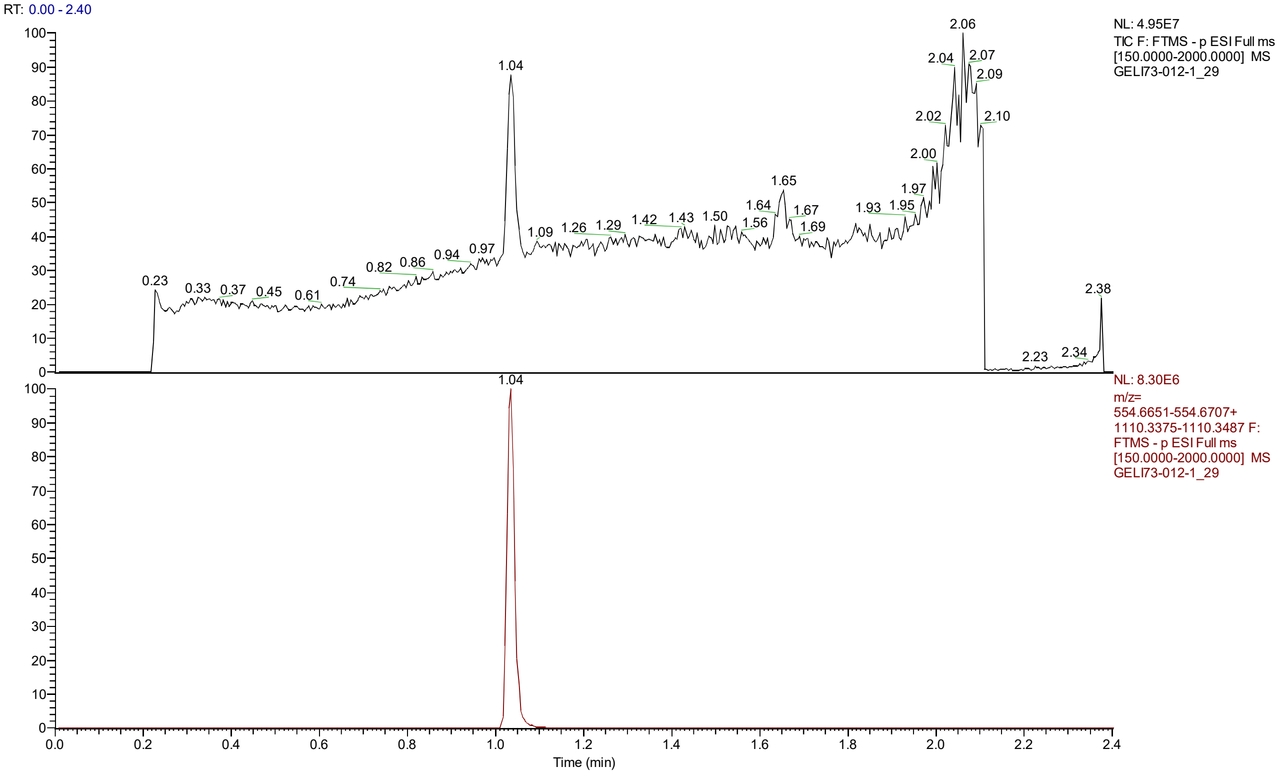

GELI73-012-1: Top: TIC, Bottom: Extracted Ion Chromatogram of m/z 554.6679.

www.symeres.com

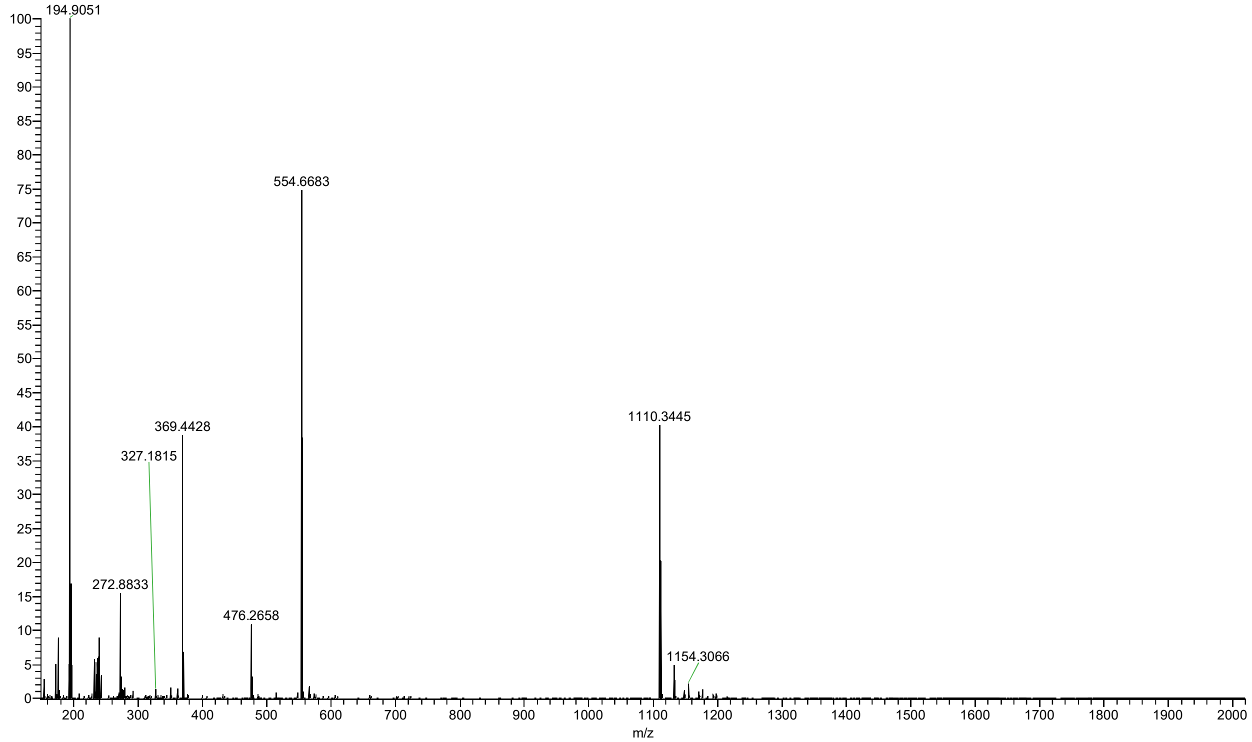

GELI-73-012-1: MS Spectrum @ 1.04 minutes.

www.symeres.com

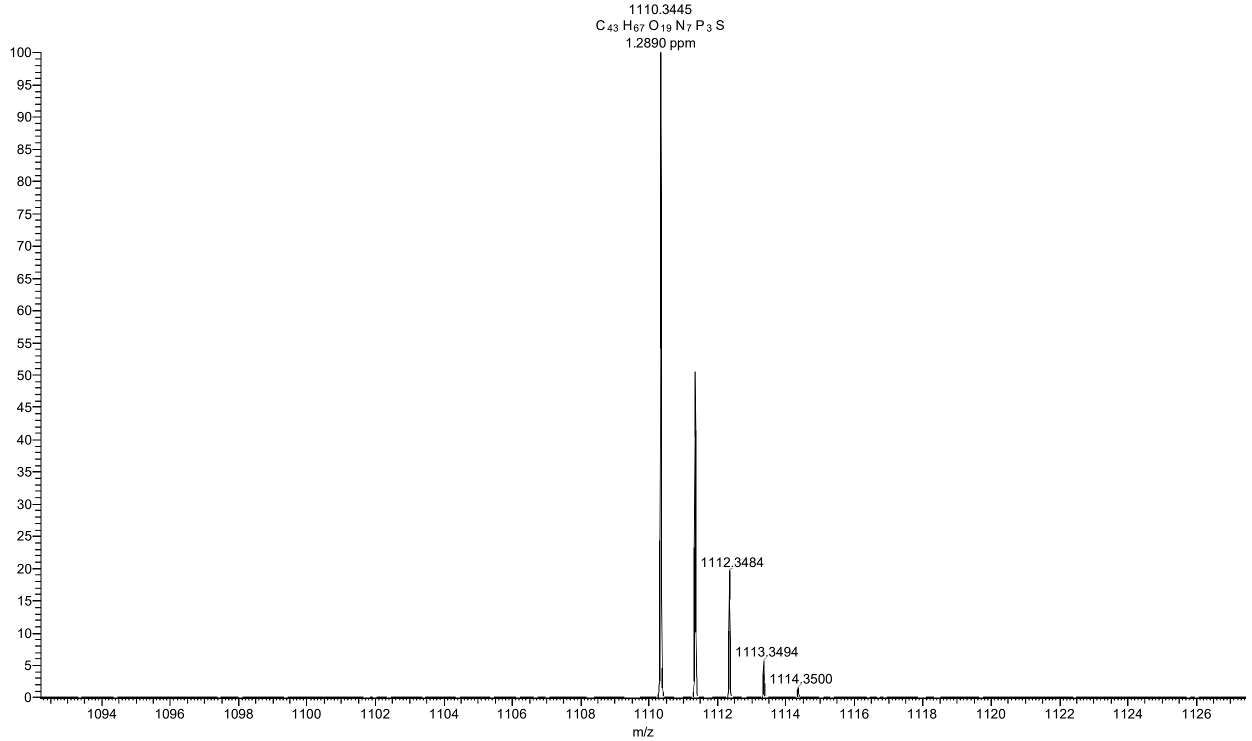

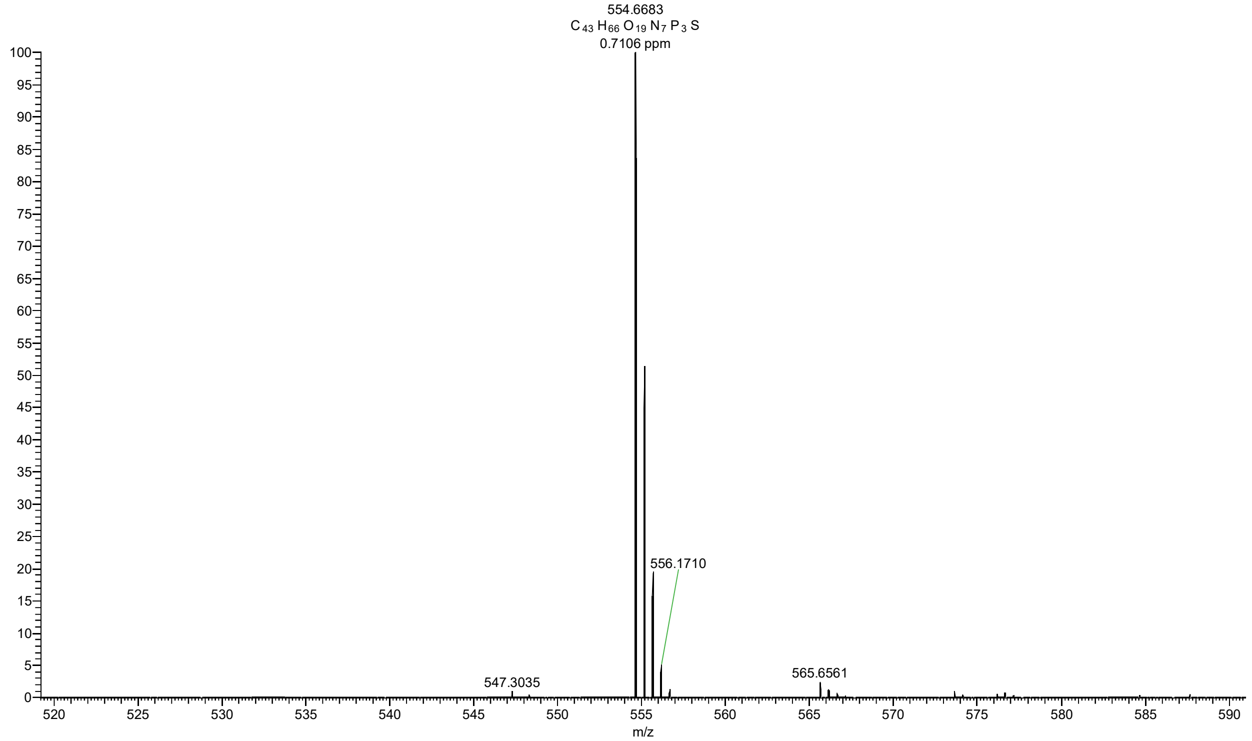

GELI-73-012-1: MS Spectrum @ 1.04 minutes, zoomed in.

www.symeres.com

3.2. GELI-015-5

HRMS (m/z): C43H68N7O19P3S, [M-H]-, Calculated: 1110.3431; found: 1110.3440, Δppm: 0.80, [M-2H]2-, Calculated: 554.6679, found 554.6680, Δppm: 0.14

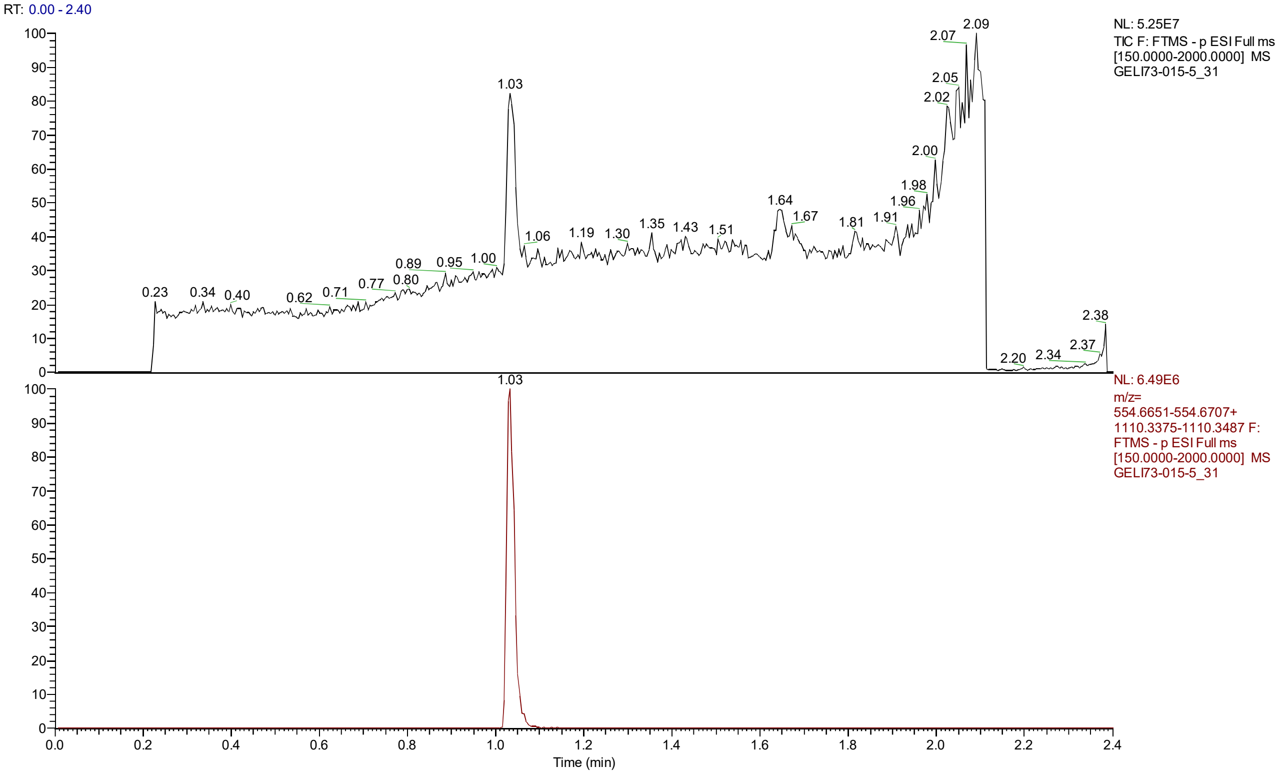

GELI73-015-5: Top: TIC, Bottom: Extracted Ion Chromatogram of m/z 1110.3431 & 554.6679

www.symeres.com

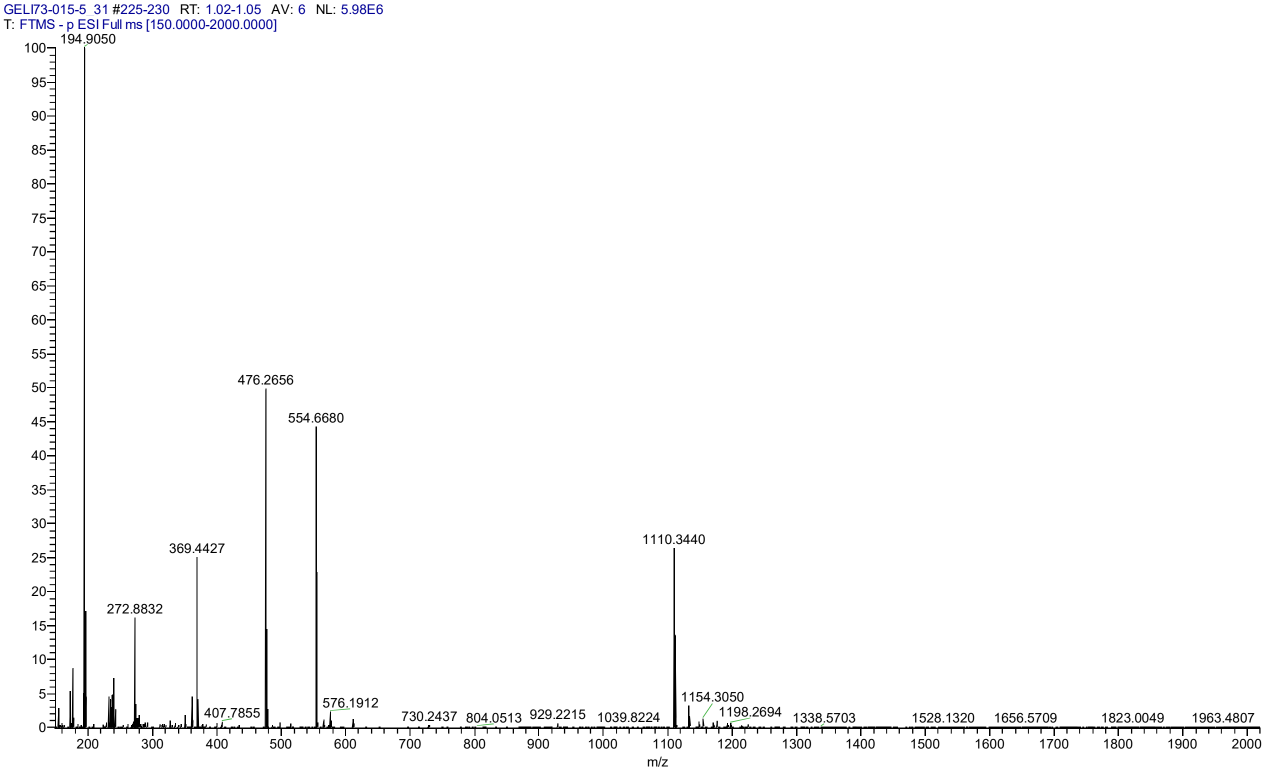

GELI-73-015-5: MS Spectrum @ 1.03 minutes.

www.symeres.com

GELI73-015-5_31 #225-230 RT: 1.02-1.05 AV: 6 NL: 1.58E6
T: FTMS - p ESI Full ms [150.0000-2000.0000]

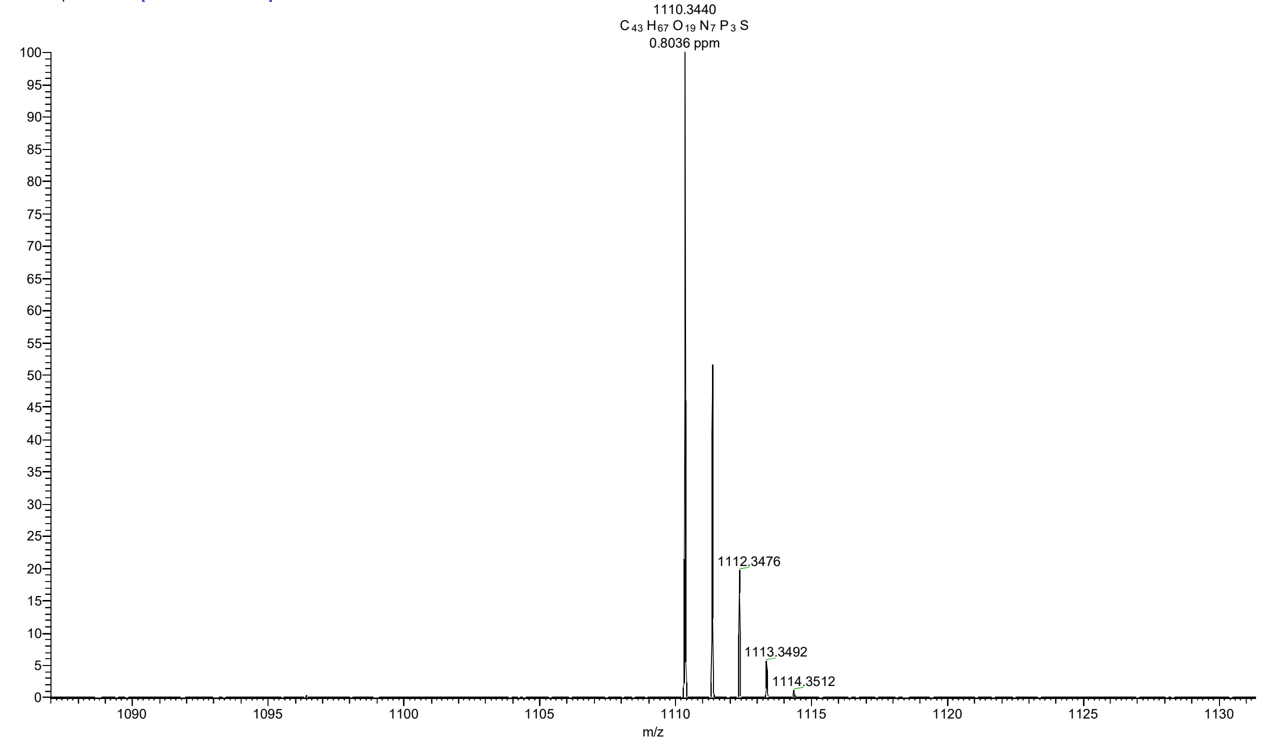

GELI73-015-5_31 #225-230 RT: 1.02-1.05 AV: 6 NL: 2.65E6
T: FTMS - p ESI Full ms [150.0000-2000.0000]

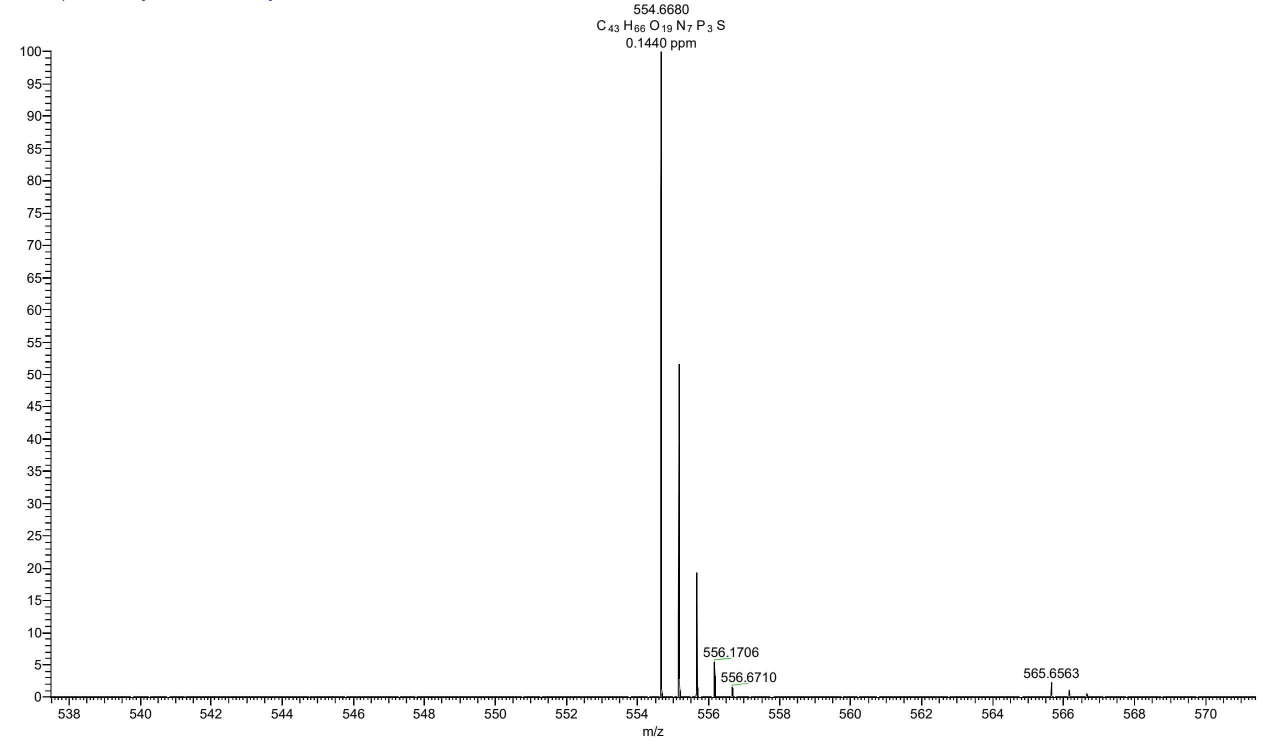

GELI-73-015-5: MS Spectrum @ 1.03 minutes, zoomed in.

www.symeres.com

4. Conclusions

The observed mass corresponds to the provided molecular formula and the calculated mass.

5. Appendix: LC-MS method used

LC-MS method (Base)

**Method name**	**HRMS Base NEG**
Mobile phase	A: 10 mM ammoniumbicarbonate (pH 9.5) in MilliQ<br>B: MeCN
Gradient elution	t (min) A(%) B(%)<br>0 95 5<br>0.2 95 5<br>1.7 5 95<br>2.1 5 95<br>2.2 95 5<br>2.4 95 5
Divert valve	0 min to waste / 0.2 min to MS / 2.1 min to waste
Flow rate	700 µl/min
Injection volume	1 µl
Sample tray temperature	6 ± 2°C
Column	Acquity CSH C18 (30x2.1, 1.7 µm)
Column temperature	35°C
Detection	Photodiode array absorbance from 220 to 500 nm<br>Electrospray mass spectrometry with full scan MS analysis (*m/z* 150-2000)

Symeres

title	**GELI73-015-5**
Method	XBridge_85-20_M1500_Deact.M
Date acquired	28-Mar-22, 18:24:48
FileName	Analysis\lcms19_20220328_0328_072_182448.d
Column	XBridge Amide (150x4.6mm, 3.5μ)
Flow	1 ml/min Column temp: 25°C
Eluent A	10 mM ammonium acetate in water (pH 9) + 2.5μM Deactivator
Eluent B	85% Acetonitrile + 15% 10mM NH4OAc in water + 2.5μM Deactiv ator
Lin. Gradient	t=0min 100% B, t=8 min 25% B, t=9min 25% B
Posttime	5 min
Detection	DAD (210-320 nm, 210 nm)
Detection	PDA (210-320 nm)
Detection	MSD (ESI pos/neg) mass range: 400-1500

PDA - Total Absorbance Chromatogram

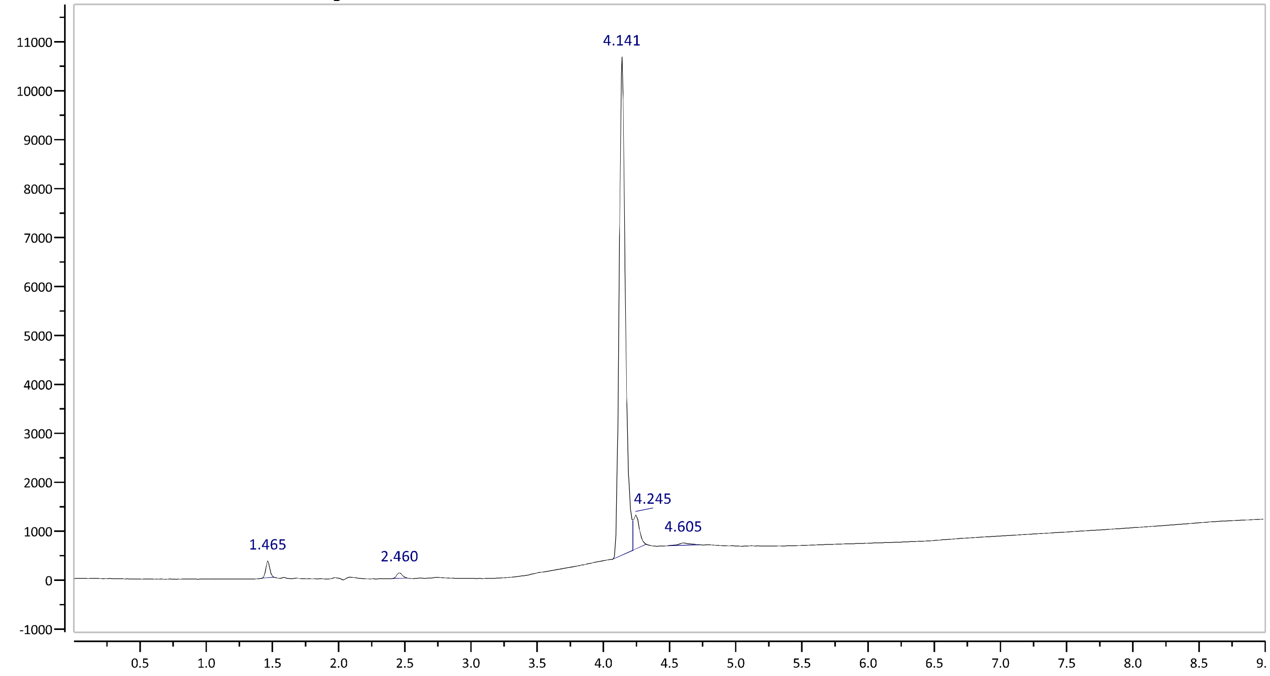

MS - spectrum 4.15..4.20

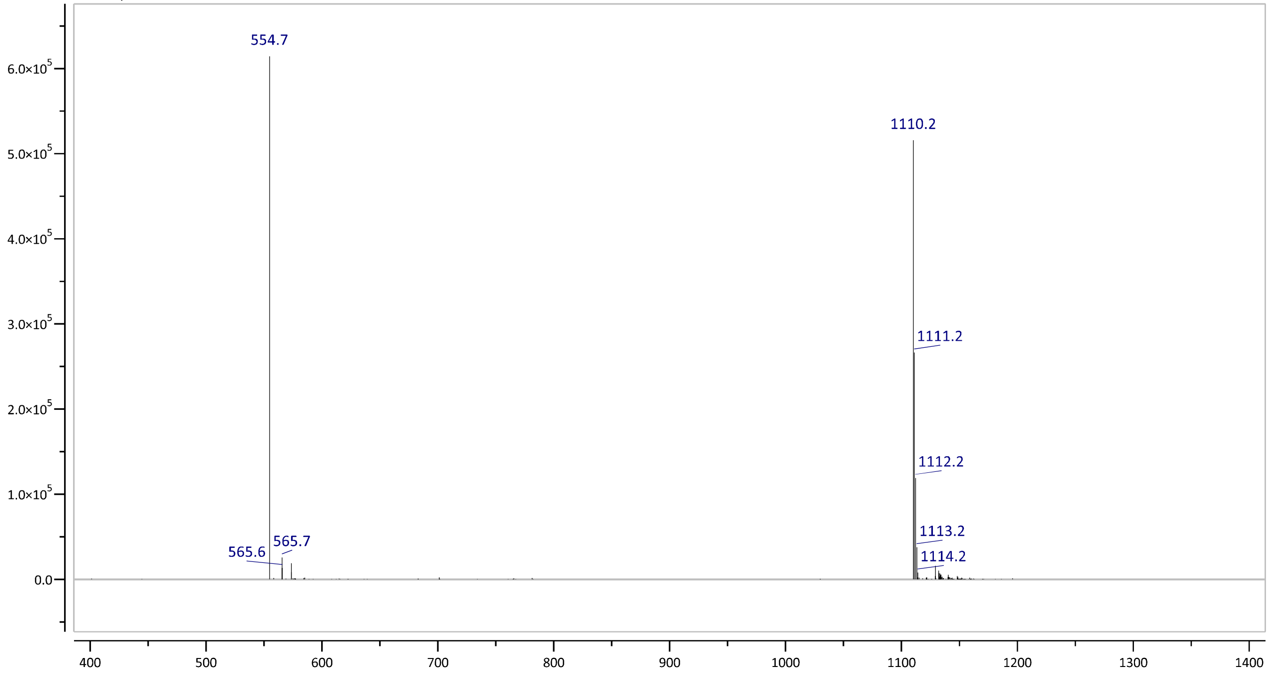

Integrals spectrum PDA - Total Absorbance Chromatogram

rt (min)	height	area	area (%)
1.46	344.0	14058	1.90
2.46	116.2	6752	0.91
4.14	10184	669585	90.54
4.24	691.9	43523	5.88
4.60	47.93	5643	0.76

Symeres

Title	**GeLi73-015-5**
Data File Name	run_20220328_0328_100834_150
Method	1D
Origin	Bruker BioSpin GmbH
Pulse Sequence	zg30
Relaxation Delay	1 s
Solvent	D2O
Acquisition Date	28 Mar 2022 (10:12)
Number of Scans	64
Frequency	400 MHz
Nucleus	1H

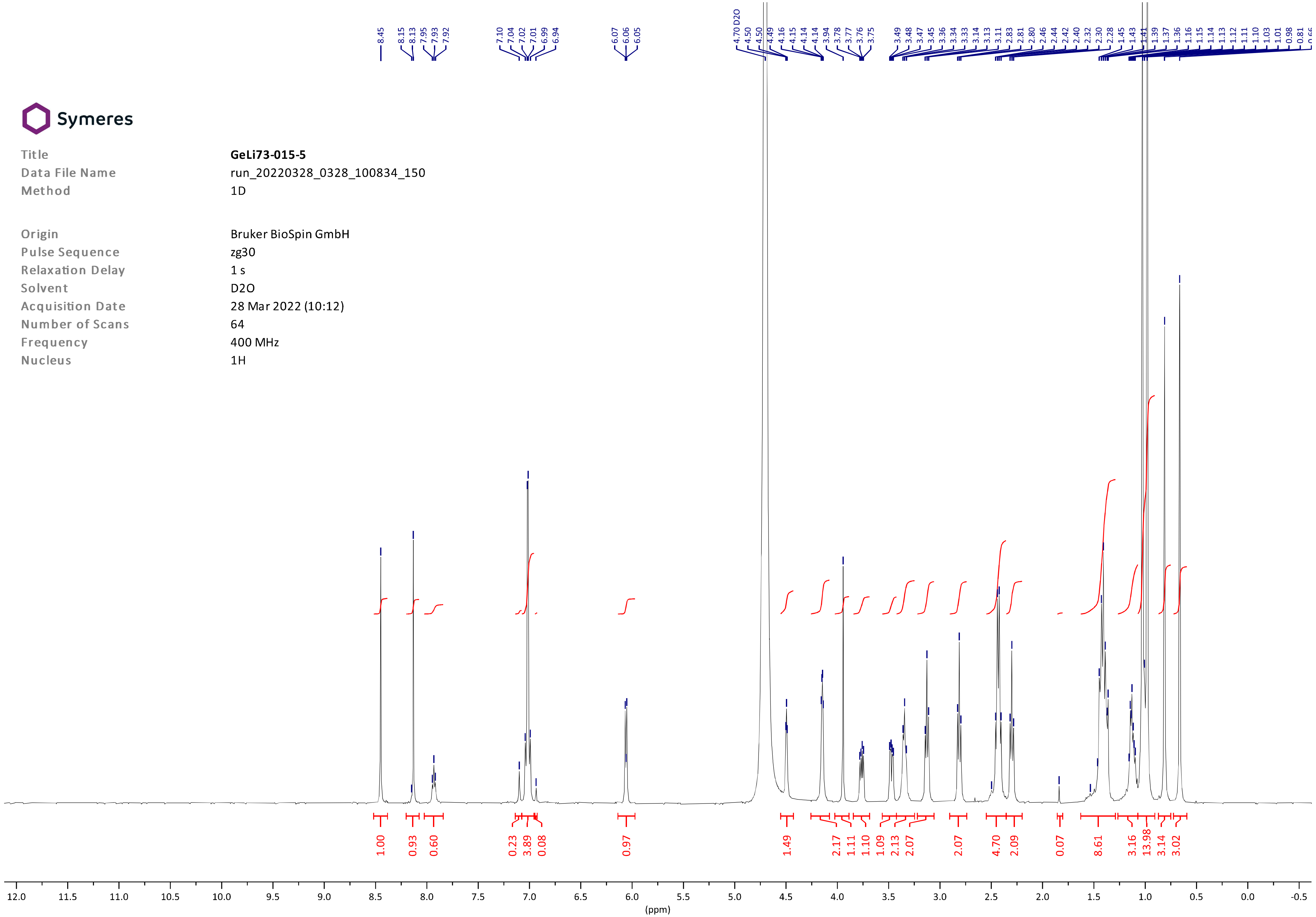

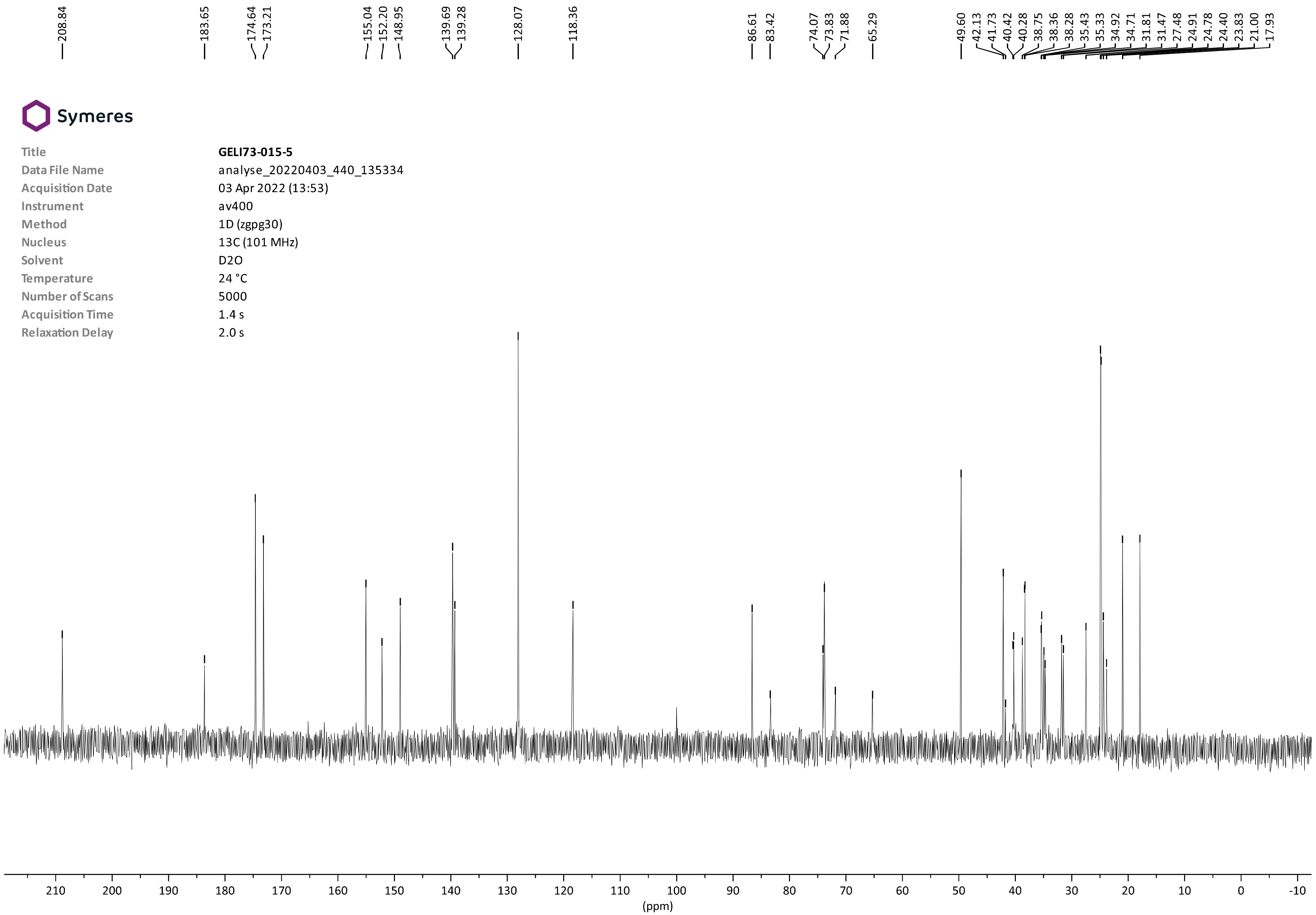

Symeres

Title	**GELI73-015-5**
Data File Name	analyse_20220403_440_135334
Acquisition Date	03 Apr 2022 (13:53)
Instrument	av400
Method	1D (zgpg30)
Nucleus	13C (101 MHz)
Solvent	D2O
Temperature	24 °C
Number of Scans	5000
Acquisition Time	1.4 s
Relaxation Delay	2.0 s

Supplementary Table 1. Effect of compounds on lipogenesis in mouse primary hepatocytes

Compound Name	Structure	% Change from control at 100 µM ±SEM(n)	Statistical difference vs control	*p*- value	IC_{50} in µM
EVT0019	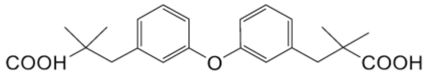	-30 ± 2.1 (n=4)	Yes	0.0020	ND
EVT0024	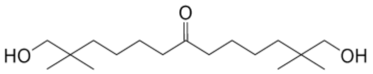	-41 ± 4.8 (n=2)	Yes	0.0229	73.23
EVT0025	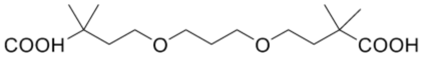	-16 ± 4.4 (n=4)	No	ns	ND
EVT0026	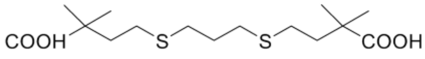	-52 ± 5.5 (n=4)	Yes	0.0073	ND
EVT0054	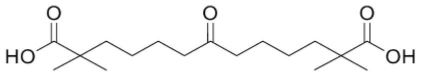	-69 ± 2.3 (n=4)	Yes	<0.0001	25.58
EVT0139	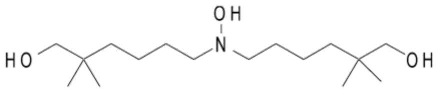	-33 ± 6.7 (n=4)	Yes	0.0151	ND
EVT0146	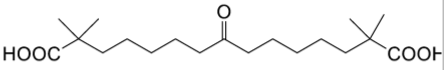	-76 ± 4.7 (n=4)	Yes	0.0014	0.46
EVT0149	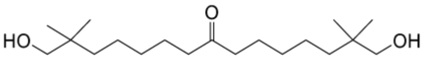	-90 ± 1.5 (n=4)	Yes	<0.0001	5.39
EVT0165	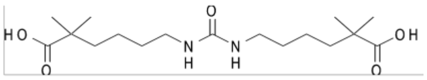	-20 ± 5.4 (n=4)	No	ns	ND
EVT0173	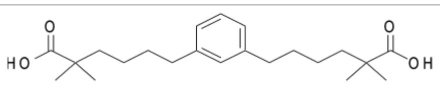	-82 ± 2.8 (n=6)	Yes	<0.0001	0.34
EVT0174	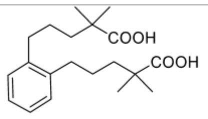	+44 ± 8.1 (n=4)	Yes	<0.0001	ND

**Compound Name**	**Structure**	**% Change from control at 100 μM ±SEM(n)**	**Statistical difference vs control**	***p*- value**	**IC$_{50}$ in μM**
EVT0175	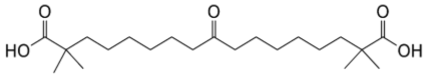	-62 ± 4.5 (n=4)	Yes	<0.0001	0.35
EVT0185	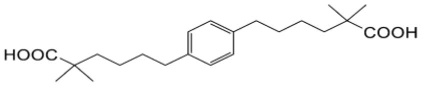	-84 ± 1.0 (n=6)	Yes	<0.0001	0.46
EVT0186	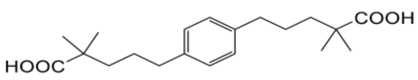	-70 ± 2.2 (n=4)	Yes	<0.0001	27.66
EVT0187	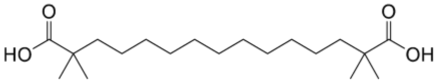	-91 ± 3.6 (n=4)	Yes	<0.0001	<0.3
EVT0199	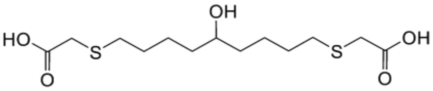	-43 ± 7.3 (n=4)	Yes	<0.0001	ND
EVT0203	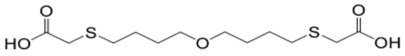	-38 ± 8.6 (n=4)	Yes	0.0091	ND
EVT0210	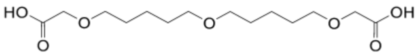	-12± 3.3 (n=4)	No	ns	ND

Supplementary Table 2. Cryo-EM data collection, refinement, and validation statistics

	EMD-53847<br>PDB ID 9R90
**Data collection and processing**	
Magnification	60,000
Voltage (kV)	300
Electron exposure (e⁻/Å^2)	61.8
Defocus range (μm)	1.2 – 1.8
Pixel size (Å)	0.72
Symmetry imposed	C1
Initial particle images (no.)	210,900
Final particle images (no.)	280,072<br>(Following symmetry expansion in D2 symmetry)
Map resolution (Å)	3.25
FSC threshold	0.143
Map resolution range (Å)	6.14 – 2.84
**Refinement**	
Initial model used	PDB 6hxh
Model resolution (Å)	3.37
FSC threshold	0.5
Model resolution range (Å)	∞ - 3.37
Map sharpening *B* factor (Å^2)	-88.8
Model composition	
Non-hydrogen atoms	14,042
Protein residues	1,800
B factors (Å^{-2})	
Protein	138.8
Ligands	108.1
R.m.s. deviations	
Bond lengths (Å)	0.003
Bond angles (°)	0.599
Validation	
MolProbity score	1.55
Clashscore	10.7
Poor rotamers (%)	0.07
Ramachandran plot	
Favored (%)	98.3
Allowed (%)	1.7
Disallowed (%)	0

Supplementary Table 3: MIBI Antibody Panel

Target	Specificity	Mass Channel	Element	Clone	Vendor	Catalogue No.	Titer (ug/mL)
dsDNA	nucleus (for segmentation)	89	Y	3519 DNA	IonPath	708901-100	1
CD19	B cell lineage	142	Nd	6OMP31	Invitrogen	14-0194-82	1
CD4	T cells	143	Nd	4SM95	IonPath	714304-100	1
CD11c	dendritic cell / macrophage	144	Nd	D1V9Y	IonPath	714402-100	1
Arginase-1		150	Nd	EPR6672(B)	IonPath	715001-100	1
CD49b	NK cell lineage	151	Eu	EPR17338	IonPath	715102-100	1
CD31	endothelial	152	Sm	D8V9E	IonPath	715202-100	1
Ki-67	proliferation	153	Eu	SP6	IonPath	715302-100	1
CD11b	macrophage	155	Gd	EPR1344	IonPath	715504-100	1
F4/80	macrophage	156	Gd	BM8	IonPath	715603-100	1
CD8	cytotoxic T cell lineage	158	Gd	CAL38	IonPath	715803-100	1
CD3e	T cell lineage	159	Tb	EPR22667-12	IonPath	715904-100	1
FAS	Fatty Acid Synthase	160	Gd	C20G5	CST	66058SF	1
ACC	Acetyl co.A Carboxylase	161	Dy	C03B10	CST	52923SF	1
Vimentin	mesenchymal	163	Dy	D21H3	IonPath	716301-100	1
alphaSMA	smooth muscle actin	164	Dy	D4K9N	IonPath	716401-100	4
PLIN-2	Perilipin-2	166	Er	Polyclonal	Novus	NB110-40877	1
B220	B cell lineage marker	167	Er	RA3-6B2	IonPath	716702-100	1
HNF4A		169	Tm	K9218	Invitrogen	MA1-199	1
CD45	lymphoid lineage	175	Lu	D3F8Q	IonPath	715503-100	1
Na-K-ATPase	membrane (for segmentation)	176	Yb	EP1845Y	IonPath	717603-100	1

Supplementary Table 4: MIBI Reagents

**Reagent**	**Vendor**	**Catalogue No.**
MIBI slides	IonPath	567001
Xylene HISTOLOGICAL GRADE	Sigma-Aldrich	534056-500
Target Retrieval Solution, pH 9, (3:1)	Agilent (Dako)	S2375
MIBI-water	IonPath	567002
low-Barium PBS	IonPath	567004
TBS-T	IonPath	567005
Normal Donkey serum	Jackson	017-000-121
ImmEdge hybrophobic barrier pen	Vector lab	H-4000
Centrifugal filters (0.1µm)	Millipore	UFC30VV00
Glutaraldehyde 8% Aqueous Solution EM Grade	EMS	16020
Tris pH8.5	IonPath	567003

Supplementary Table 5: MIBI software

**Resource**	**Source**
MIBI/O 1.11.1	IonPath
Data analysis post image processing were done using MATLAB 2021b and R Studio 4.2.0	Mathworks and cran.r-project.org
Mesmer	deepcell.org
FlowSOM	Bioconductor R package
cytomapper	Bioconductor R package
Spectre	R package
CATALYST	Bioconductor R package
CytoMAP	MATLAB GUI software

Supplementary Figure 1

Extended Figure 10g

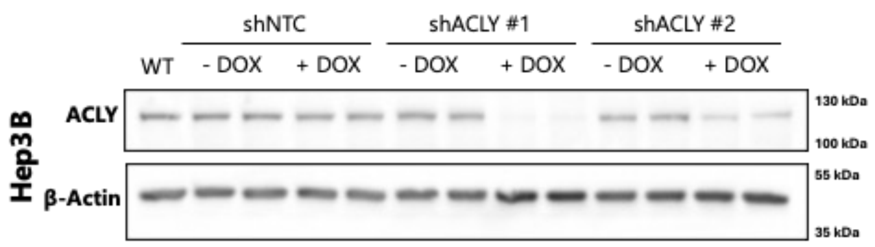

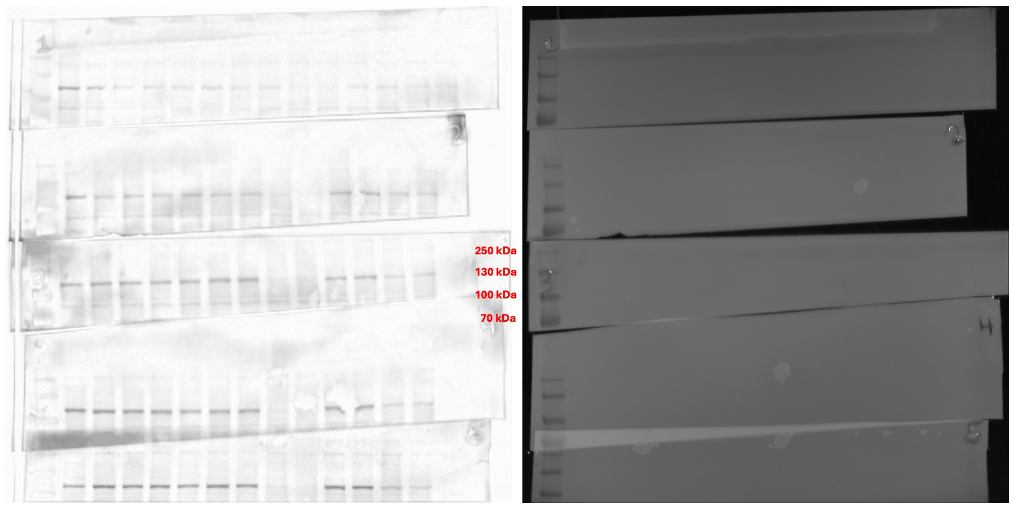

ACLY blots 1 to 5
Exposure 14.3 seconds

Blot 3 was selected

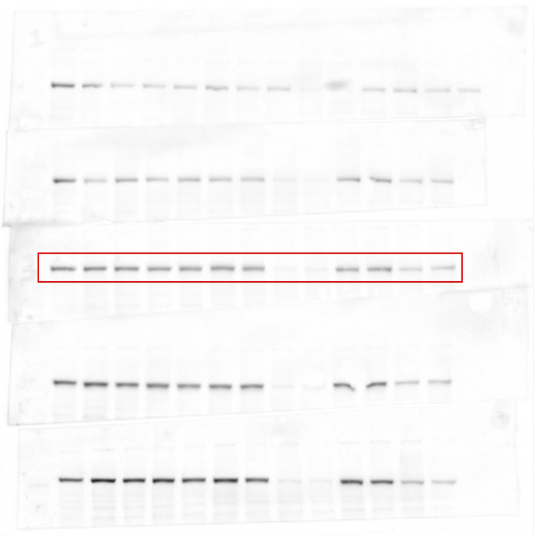

ACLY blots 1 to 5
Supersignal
Exposure 2.2 seconds

Blot 3 was selected

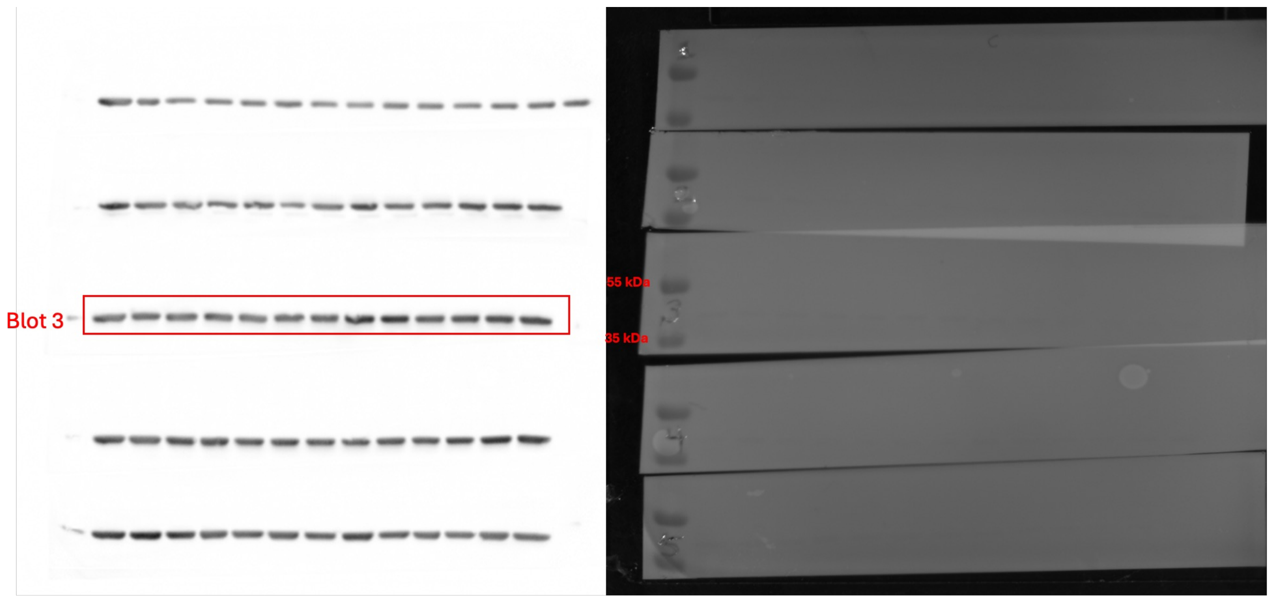

β-Actin blots 1 to 5
Exposure 0.4 second

1. The blots selected are highlighted in red.
2. Loading control, β-Actin was run on the same gel as of protein of interest (ACLY)

Extended Figure 3d, 3e

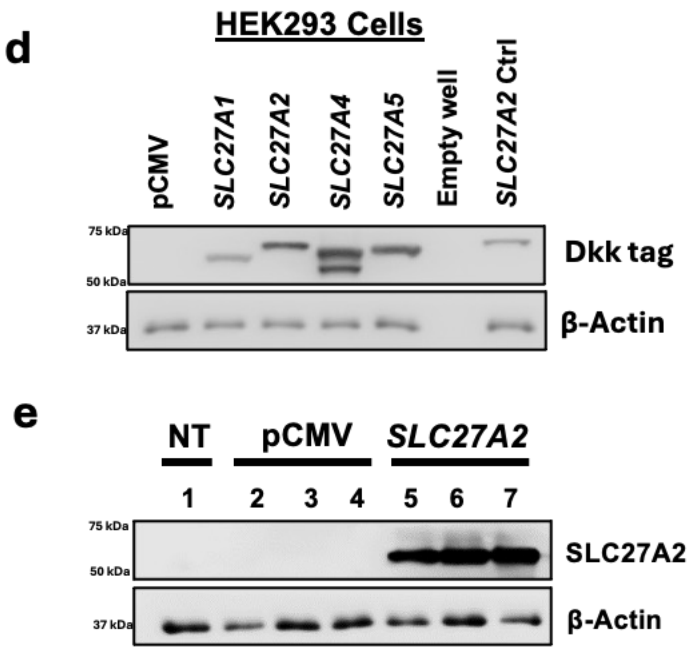

Extended Data Figure 3d

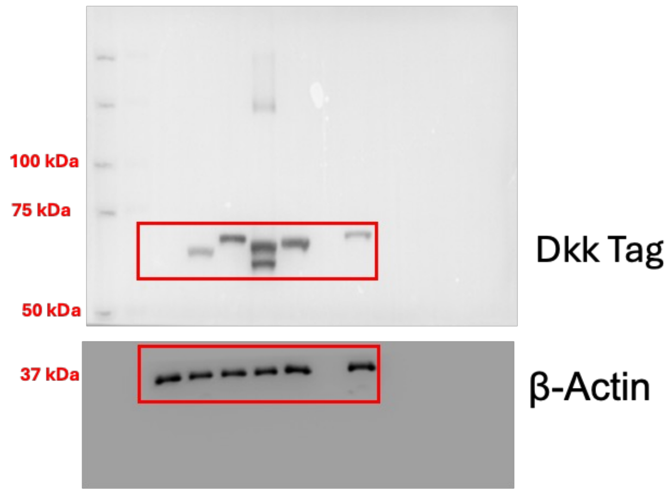

Extended Data Figure 3e

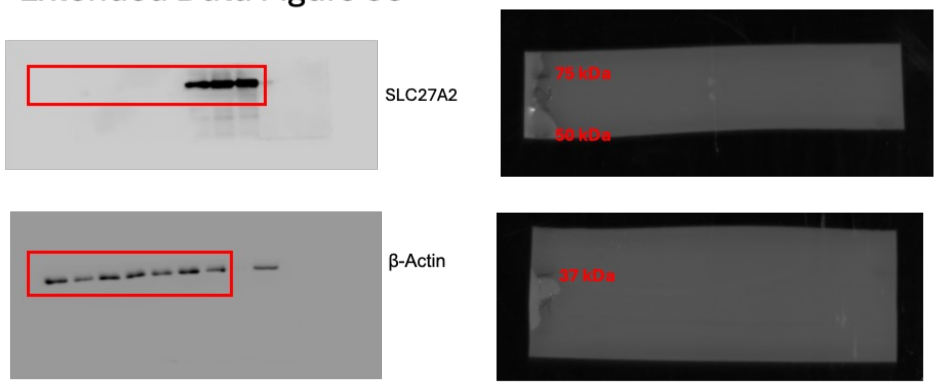

1. The blots selected are highlighted in red.
2. Loading control, β-Actin was run on the same gel as of protein of interest (Dkk Tag or SLC27A2).
