# Supplementary material for: ACLY inhibition promotes tumour immunity and suppresses liver cancer
Source: Nature. 2025 Jul 30;645(8080):507–17. doi: 10.1038/s41586-025-09297-0 (PMC12422966; doi:10.1038/s41586-025-09297-0)
Supplement: Supplementary file 2 — Reporting Summary [file 41586_2025_9297_MOESM2_ESM.pdf]

nature portfolio

Corresponding author(s): Dr. Gregory R. Steinberg

Last updated by author(s): May 15, 2025

Reporting Summary

Nature Portfolio wishes to improve the reproducibility of the work that we publish. This form provides structure for consistency and transparency in reporting. For further information on Nature Portfolio policies, see our Editorial Policies and the Editorial Policy Checklist.

Statistics

For all statistical analyses, confirm that the following items are present in the figure legend, table legend, main text, or Methods section.

n/a	Confirmed	
☐	☒	The exact sample size (*n*) for each experimental group/condition, given as a discrete number and unit of measurement
☐	☒	A statement on whether measurements were taken from distinct samples or whether the same sample was measured repeatedly
☐	☒	The statistical test(s) used AND whether they are one- or two-sided<br>*Only common tests should be described solely by name; describe more complex techniques in the Methods section.*
☐	☒	A description of all covariates tested
☐	☒	A description of any assumptions or corrections, such as tests of normality and adjustment for multiple comparisons
☐	☒	A full description of the statistical parameters including central tendency (e.g. means) or other basic estimates (e.g. regression coefficient) AND variation (e.g. standard deviation) or associated estimates of uncertainty (e.g. confidence intervals)
☐	☒	For null hypothesis testing, the test statistic (e.g. *F*, *t*, *r*) with confidence intervals, effect sizes, degrees of freedom and *P* value noted<br>*Give P values as exact values whenever suitable.*
☒	☐	For Bayesian analysis, information on the choice of priors and Markov chain Monte Carlo settings
☒	☐	For hierarchical and complex designs, identification of the appropriate level for tests and full reporting of outcomes
☐	☒	Estimates of effect sizes (e.g. Cohen's *d*, Pearson's *r*), indicating how they were calculated

Our web collection on statistics for biologists contains articles on many of the points above.

Software and code

Policy information about availability of computer code

Data collection

Flow cytometry data were collected using CytoFlex (Beckman Coulter Life Sciences) and BD LSR Fortessa (BD Biosciences).

Immunofluorescence data were collected on an inverted confocal microscope (Leica Microscope Systems).

MIBI : Spectral images of stained liver lesions were collected using an Ionpath MIBIscope with Multiplexed Ion-Beam Imaging technology. For Acly Ko cohort, thirty-five 400x400µm regions for each experimental group, representing 43,974 cell objects were acquired while for EVT0185 cohort, twenty-four 400x400µm regions for each experimental group were collected for a total of 30,191 cell objects.

Bulk RNA seq : Next-generation sequencing was conducted at the McMaster Genomics Facility, Farncombe Institute, McMaster University, using Illumina HiSeq 1500 (Illumina; San Diego, CA, USA). Samples were randomly distributed across lanes of a HiSeq Rapid v2 flow cell to eliminate lane-specific effects and single-end 50 bp reads were generated at 12.5 million clusters per sample.
Microarray CEL files were downloaded from GSE164760.
Raw RNA-Seq files for tumor samples derived from DEN and CCl4 models from PRJNA488497 and PRJNA386995 respectively.

ScRNA seq: Sequencing was performed using the Illumina Novaseq 6000 sequencing system (S4, 2x150, 2-2.5 billion reads, targeting 5000 cells per sample at a depth of 25000 read pairs per cell).

Single nuclei sequencing: Sequencing of cells derived from Healthy, MASLD, MASH-HCC tumor and tumor adjacent tissue were obtained from GSE174748 and GSE189175

Spatial Transcriptomics: Slides with 5 µm of tissue sections were processed with deparaffinization, H&E staining, imaging, decrosslinking,

hybridization, ligation, probe extension, pre-amplification and probe-based library construction to generate gene expression libraries for each tissue for following sequencing. Images were taken using the Nikon 90i Eclipse upright microscope. RNA-seq was performed using the Illumina NextSeq 2000 (P2 Flow cell, 2×50bp configuration) system.

Grids were screened using a JEOL 1400 Plus microscope equipped with a JEOL Ruby CCD camera at the VIB Bioimaging Core Ghent. Cryo-electron microscopy data were collected at the VIB-VUB facility for Bio Electron Cryogenic Microscopy (BECM, Brussels, Belgium)

Data analysis

Flow cytometry: FlowJo v10.8.1

Immunofluorescence and lipid droplet analysis: Image J.

CD3 and CD19 positive cells counting: Halo software.

MIBI analysis: Segmentation mask : Mesmer, Single cell phenotyping:'FlowSOM' R package
Cellular Neighborhood analyiss: ImcRtools package

RNA seq analysis: Sequence quality: FastQC , Removal of low-quality reads and adapter sequences: Cutadapt, Genome alignment: HISAT2, Quantification of reads: Feature Counts, Surrogate variable analysis: sva v3.44.0, Differential gene expression analysis: DESeq2 package v1.36.0, Differential expression analysis for microarray data: limma v3.52.3, Over-representation and GSEA : clusterProfiler v4.4.4, Semantic similarity and binary cut method: simplifyEnrichment v1.6.1, Murine cell-type deconvolution: transcript-per-million (TPM) normalized gene expression data and mMCP-counter v1.1.0., Human cell-type deconvolution: TIMER2.0 and ESTIMATE package v1.0.13.

ScRNA seq analysis: Demultiplexing-Cell Ranger, Integration analysis-Seurat v5,Mouse-human correlation analysis-biomaRt package, Human gene-specific mean expression data-Seurat v5 in R studio.

Single nuclei sequencing: Processed using the Seurat package version 5.1.0 in R, Cell type identification-sc-type package in R.

Spatial Transcriptomics: Sequence data from FFPE tissues- Space Ranger count pipeline from 10X genomics, Quality control, normalization, dimensional reduction and clustering, variable gene selection, spatially-variable feature detection, annotation, differential expression, integration with multiple samples- Seurat, Spatial Transcriptomics data analyses- Linux system, R, RStudio software and Python programming language

Single-particle cryo-electron microscopy data analysis- CryoSparc v3.1.0. Neural-network based particle picking- TOPAZ. The atomic model for human ACLY (pdb 6hxh)- fitted ChimeraX and Coot and real-space refined in Phenix. Structural analysis-Pymol. Restraints for EVT0185-CoA -de Grade Web Server (https://grade.globalphasing.org).

For manuscripts utilizing custom algorithms or software that are central to the research but not yet described in published literature, software must be made available to editors and reviewers. We strongly encourage code deposition in a community repository (e.g. GitHub). See the Nature Portfolio guidelines for submitting code & software for further information.

Data

Policy information about availability of data

All manuscripts must include a data availability statement. This statement should provide the following information, where applicable:

- Accession codes, unique identifiers, or web links for publicly available datasets
- A description of any restrictions on data availability
- For clinical datasets or third party data, please ensure that the statement adheres to our policy

Data that support the findings of this study are available within the article and its Supplementary information. The bulk RNA-seq data of the WD-DEN liver tumors, and spatial transcriptomic data of the WD-DEN and WD-CCl4 livers have been deposited at NCBI Gene Expression Omnibus (GEO) and are accessible under accession numbers GSE296668 and GSE297081, respectively. For the mouse HCC model comparision, raw RNA-Seq files for tumor samples derived from Control-DEN and WD-CCl4 models were downloaded from the NCBI Sequence Read Archive under reference numbers PRJNA488497 and PRJNA386995 respectively. Single-nuclei sequencing of cells derived from Healthy, MASLD, MASH-HCC tumor and tumor adjacent tissue were obtained from GSE174748 and GSE189175. Gel source data are provided in Supplementary Figure 1. Source data are provided with this paper.

Cryo-EM maps following global and local refinement and the real-space refined model for the CCS/CSH assembly have been deposited in the Electron Microscopy Data Bank (EMDB) and Protein Data Bank with accession codes EMDB 17148 and PDB 8OS4. Detail info is in Supplementary Table S2.

Research involving human participants, their data, or biological material

Policy information about studies with human participants or human data. See also policy information about sex, gender (identity/presentation), and sexual orientation and race, ethnicity and racism.

Reporting on sex and gender	Not Applicable
Reporting on race, ethnicity, or other socially relevant groupings	Not Applicable
Population characteristics	Not Applicable

Recruitment	Not Applicable
Ethics oversight	Not Applicable

Note that full information on the approval of the study protocol must also be provided in the manuscript.

Field-specific reporting

Please select the one below that is the best fit for your research. If you are not sure, read the appropriate sections before making your selection.

☒ Life sciences ☐ Behavioural & social sciences ☐ Ecological, evolutionary & environmental sciences

For a reference copy of the document with all sections, see nature.com/documents/nr-reporting-summary-flat.pdf

Life sciences study design

All studies must disclose on these points even when the disclosure is negative.

Sample size	Sample size was calculated based on the animal availability after around 7 months of DEN injection. The sample size were considered as adequate based on the previous experiments which would be sufficient to provide meaningful conclusions (Cell metabolism. 2019 Jan 8;29(1)). No statistical method was used to predetermine sample size.
Data exclusions	No data were excluded during the analysis
Replication	Acly KO Animal experiments were performed three times and replication attempt was successful.
Randomization	Mice were randomized based on their serum AFP (Alpha Feto protein) levels prior to the treatment/ AAV injection
Blinding	Animal experiments were not blinded. However, tissue collection, histological, biochemical analysis and other analytic tests were blinded.

Reporting for specific materials, systems and methods

We require information from authors about some types of materials, experimental systems and methods used in many studies. Here, indicate whether each material, system or method listed is relevant to your study. If you are not sure if a list item applies to your research, read the appropriate section before selecting a response.

Materials & experimental systems

n/a	Involved in the study	
☐	☒	Antibodies
☐	☒	Eukaryotic cell lines
☒	☐	Palaeontology and archaeology
☐	☒	Animals and other organisms
☒	☐	Clinical data
☒	☐	Dual use research of concern
☒	☐	Plants

Methods

n/a	Involved in the study	
☒	☐	ChIP-seq
☐	☒	Flow cytometry
☒	☐	MRI-based neuroimaging

Antibodies

Antibodies used

Western Blot: SLC27A2 (Invitrogen, # PA5-30420), B-Actin (Cell Signaling Technology, #X5125S)

Immunofluorescense microscopy: CCP3 (Cell Signaling Technology, #9664S), Ki67 (ThermoFisher, #MA5-14520)

MIBI imaging: dsDNA nucleus DNA (IonPath, # 708901-100), CD19 (Invitrogen, #14019482), CD4 (Ionpath, #714304-100), CD11c (IonPath, #714402-100), Arginase-1 (IonPath, # 715001-100), CD49b (IonPath, # 715102-100), CD31 e (IonPath, # 715202-100), Ki-67 (IonPath, # 715302-100), CD11b (IonPath, #715504-100), F4/80 (IonPath, # 715603-100), CD8 (IonPath, # 715803-100), CD3e (IonPath, # 715904-100), FAS (CST, #66058SF) , ACC (IonPath, #52923SF), Vimentin (IonPath, #716301-100), alphaSMA (IonPath, #716401-100), PLIN2 (Novus, # NB110-40877), B220 (IonPath, #716702-100), HNF4A (Invitrogen, MA1-199), CD45 (IonPath, # 715503-100), Na-K-ATPase membrane (IonPath, #717603-100).The antibodies are listed in Supplementary Table S3 as well.

Flow cytometry: Human recombinant IL-2 (Preprotech, #200-02), R848 (mAb tech, #36611), E780 Live/Dead stain (Thermofisher, #C34570), Anti-human CD3 (Biolegend, #300434), Anti-human CD19 (Biolegend, #302241), CD45.2 BV510 (BioLegend, #109838), B220 (BD Biosciences, #563894), CD19 (Biolegend, #152409) and 7AAD (Thermo Fisher Scientific, #A1310).

B cell depletion study: Isotype control (Biolegend, #400566) and Anti-CD20 antibody (Biolegend, #152104)

Validation	All antibodies applicable for western blot, Immunofluorescence microscopy, Flow cytometry and MIBI imaging were validated by manufacturers and validation report is available in the corresponding website.

Eukaryotic cell lines

Policy information about cell lines and Sex and Gender in Research

Cell line source(s)	Mouse primary hepatocytes- Male C57BL-6/J mouse<br>HEK-293-ATCC<br>Hep3B-ATCC<br>Hepa1-6-ATCC
Authentication	Cell lines have been authenticated by original source and are authenticated in-house by observation of cell morphology
Mycoplasma contamination	Cell lines were not tested for mycoplasma contamination
Commonly misidentified lines (See ICLAC register)	No commonly misidentified cell lines were used

Animals and other research organisms

Policy information about studies involving animals; ARRIVE guidelines recommended for reporting animal research, and Sex and Gender in Research

Laboratory animals	C57BL-6/Acly f/f, C57BL-6 mice were used for animal experiments
Wild animals	None
Reporting on sex	Male mice were used for animal experiments. Sex was not considered during the experiments.
Field-collected samples	The study did not involve samples collected from the field.
Ethics oversight	Animal experiments were carried out using the guidelines approved by the Animal Research Ethics Board at McMaster University, Canada (Steinberg Laboratory Animal Utilization Protocol #16-12-42, 21-01-04) or the Institutional Animal Care and Use Committee (IACUC) at Icahn School of Medicine at Mount Sinai, NY (IACUC approval# PROTO202100080).

Note that full information on the approval of the study protocol must also be provided in the manuscript.

Plants

Seed stocks	Not applicable
Novel plant genotypes	Not applicable
Authentication	Not applicable

Flow Cytometry

Plots

Confirm that:

- [x] The axis labels state the marker and fluorochrome used (e.g. CD4-FITC).
- [x] The axis scales are clearly visible. Include numbers along axes only for bottom left plot of group (a 'group' is an analysis of identical markers).
- [x] All plots are contour plots with outliers or pseudocolor plots.
- [x] A numerical value for number of cells or percentage (with statistics) is provided.

Methodology

Sample preparation	Peripheral Blood Mononuclear Cells (PBMCs) were isolated from donors using the Ficoll Paque density gradient method.

Sample preparation	PBMCs were then washed, resuspended in complete RPMI1640 (10% FBS, 1% P/S, 1% L-Glutamine), and plated in 12 well plates. Next, PBMCs were incubated at 37oC, 5% CO2 incubator for 5 days. Cells were then harvested, stained with E780 Live/Dead stain (Thermofisher, #C34570), CD3 (Biolegend, #300434), CD19 (Biolegend, #302241) antibody and analyzed using flow cytometry.<br>For B cell depletion confirmation, the tail vein blood was collected from each animal after 15 days of single injection with 250 µg of anti-CD-20 (Biolegend, 152104) or isotype (Biolegend, 400566) antibody. The Red blood cells were lysed twice with 1X RBC lysis buffer. The samples were then centrifuged at 1500 rpm for 5 min at 4 °C. The cells were washed and blocked with Fc block (BD Biosciences, 553142) and stained with CD45.2 BV510 (BioLegend, 109838), B220 (BD Biosciences, 563894), CD19 (Biolegend, 152409) and 7AAD (Thermo Fisher Scientific, A1310).
Instrument	CytoFlex (Beckman Coulter Life Sciences) and BD LSR Fortessa (BD Biosciences).
Software	FlowJo v10.8.1
Cell population abundance	Cells were not sorted in the present study.
Gating strategy	For PBMC experiment,FSC vs SSC plot of lymphocytes population, FSC-A vs FSC-H to identify single cell population, FSC vs e780 Viability Dye to identify live cells population, CD19 vs CD3 to identify CD19+ B cells and CD3+ T cells.<br><br>For B cell depletion study, SSC vs FSC plot of lymphocytes population, FSC-H vs FSC-A to identify single cell population, FSC vs 7-AAD to identify live cells population, B220 vs CD19 to identify B220+CD19+ B cell population

☒ Tick this box to confirm that a figure exemplifying the gating strategy is provided in the Supplementary Information.
