# Supplementary figures and images for: ACLY inhibition promotes tumour immunity and suppresses liver cancer
Source: Nature. 2025 Jul 30;645(8080):507–17. doi: 10.1038/s41586-025-09297-0 (PMC12422966; doi:10.1038/s41586-025-09297-0)

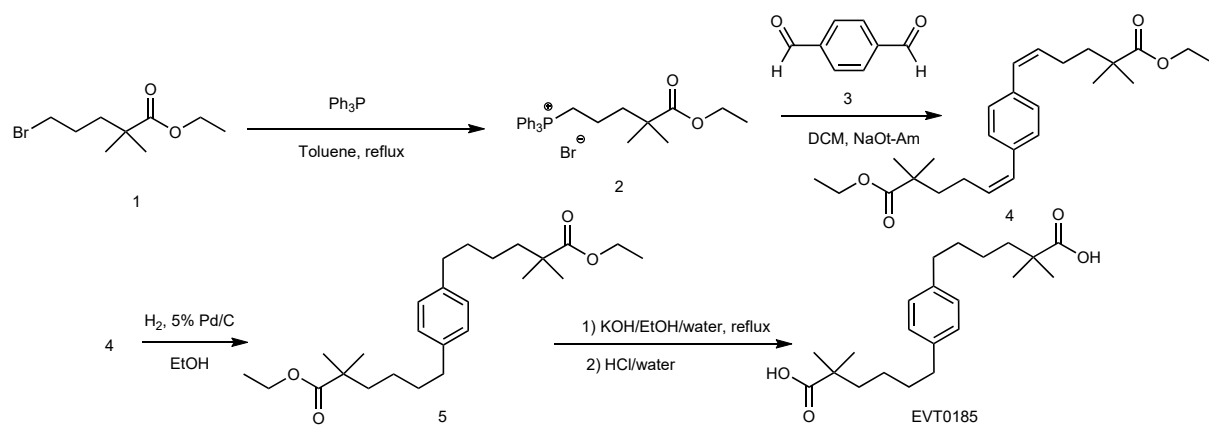

Supplement: Supplementary file 3 — Scheme 1: synthetic pathway EVT0185 [file 41586_2025_9297_MOESM3_ESM.pdf]

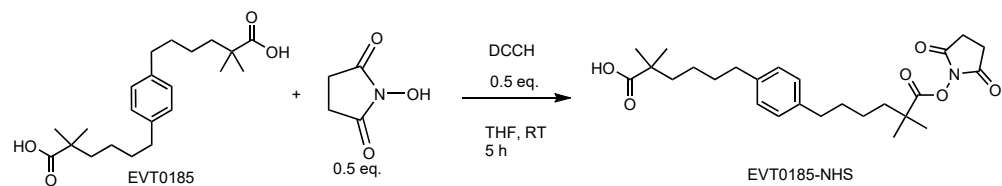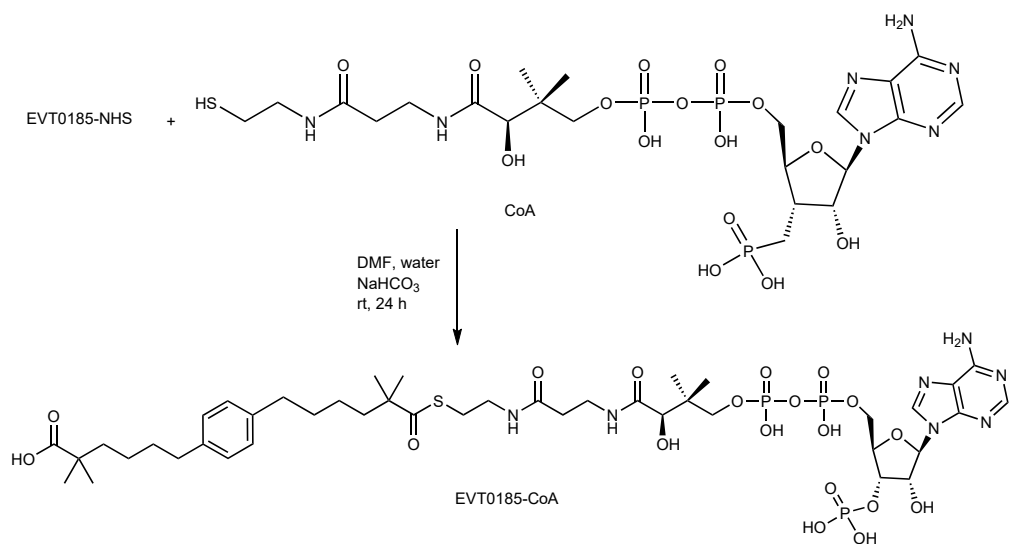

Supplement: Supplementary file 4 — Scheme 2: synthetic pathway EVT01850_CoA [file 41586_2025_9297_MOESM4_ESM.pdf]

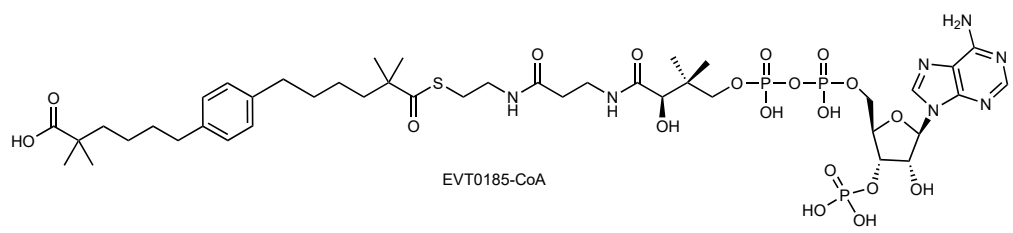

Supplement: Supplementary file 5 — Structure EVT0185 CoA [file 41586_2025_9297_MOESM5_ESM.pdf]

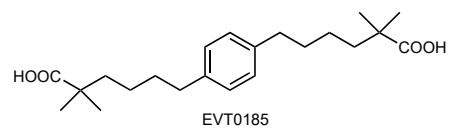

Supplement: Supplementary file 6 — Structure EVT0185 [file 41586_2025_9297_MOESM6_ESM.pdf]
